# Respiratory adverse effects in patients treated with immune checkpoint inhibitors in combination with radiotherapy: a systematic review and meta-analysis

**DOI:** 10.1186/s13014-024-02489-4

**Published:** 2024-10-01

**Authors:** Zhongjun Ma, Jiexuan Hu, Fei Wu, Naijia Liu, Qiang Su

**Affiliations:** 1 Department of Oncology, Beijing Friendship Hospital, Capital Medical University, Beijing, 100050 China; 2 National Institute for Nutrition and Health, Chinese Center for Diseases Control and Prevention, Beijing, China

**Keywords:** Immune checkpoint inhibitors, Radiotherapy, Respiratory adverse effects, Solid tumors, Meta-analysis

## Abstract

**Background:**

We conducted a systematic review and meta-analysis to assess the risk of respiratory adverse effects in patients with solid tumors treated with immune checkpoint inhibitors (PD-1, PD-L1 and CTLA-4 inhibitors) in combination with radiation therapy.

**Methods:**

We selected eligible studies through the following databases: PubMed, Embase, Cochrane Library, and Clinicaltrials (https://clinicaltrials.gov/). The data was analyzed by using Rstudio.

**Results:**

Among 3737 studies, 26 clinical trials, including 2670 patients, were qualified for the meta-analysis. We evaluated the incidence rates of adverse respiratory events, including cough, pneumonia, upper respiratory tract infections, and others: grades 1–5 cough, 0.176 (95%CI: 0.113–0.274, I2 = 92.36%); grades 1–5 pneumonitis, 0.118 (95%CI: 0.067–0.198, I2 = 88.64%); grades 1–5 upper respiratory tract infection, 0.064 (95%CI: 0.049–0.080, I2 = 0.98%); grades 3–5 cough, 0.050 (95%CI: 0.012–0.204, I2 = 8.90%); grades 3–5 pneumonitis, 0.052 (95%CI: 0.031–0.078, I2 = 83.86%); grades 3–5 upper respiratory tract infection, 0.040 (95%CI: 0.007–0.249, I2 = 45.31%).

**Conclusions:**

Our meta-analysis demonstrated that ICI combined with radiotherapy for solid tumors can produce respiratory adverse effects. ICIs combination treatment, a tumor located in the chest, is more likely to cause adverse reactions, and SBRT treatment and synchronous treatment will bring less incidence of adverse reactions. This study provide insights for clinicians to balance the risks of radiotherapy in the course of treating oncology patients.

**Supplementary Information:**

The online version contains supplementary material available at 10.1186/s13014-024-02489-4.

## Highlights

- Immune checkpoint inhibitors combined with radiotherapy can cause adverse lung effects during the treatment of cancer patients.
- Cough and pneumonia are more common pulmonary adverse reactions that deserve clinicians’ attention.
- The combination treatment of PD-1 inhibitors and PD-L1  inhibitors, tumor location in the lung are more likely to cause pulmonary adverse reactions.

**Supplementary Information:**

The online version contains supplementary material available at 10.1186/s13014-024-02489-4.

## Introduction

Immune checkpoint inhibitors (ICIs) have made a dramatic difference in the treatment of cancer, and the clinical prognosis of many cancer patients has benefited from them. ICIs targeting cytotoxic T lymphocyte-associated 4 (CTLA-4), programmed cell death protein 1 (PD-1), and PD ligand 1 (PD-L1) inhibit their activity to stop cancer cells from escaping T-cell-mediated death [1].

Nowadays, radiotherapy is frequently employed to treat cancers, nearly half of all cancer patients indicate radiotherapy [2]. Modern radiotherapy techniques include intensity-modulated radiation therapy (IMRT), stereotactic body radiation therapy (SBRT), volumetric modulated arc therapy (VMAT), and so on. They irradiate tumors with radiation to control and kill them through local treatment. In addition, emerging technologies such as Yttrium-90 radioembolization have been used to treat cancer in recent years [3].

As early as 2012, an investigator published a case report about leptomeningeal melanoma’s clinical and radiological response after the sequential treatment of whole-brain radiotherapy and ICI (Ipilimumab) [4]. With increased study and technological advancement, the application of ICIs combined with radiotherapy for solid tumors is progressively gaining attention. Radiotherapy can affect systemic immunity through abscopal effects [5], which means radiotherapy induces immune cell death and leads to the production and release of damage-associated molecular patterns (DAMPs) in the mesenchyme of tumor tissues, which promotes the immune system to increase the release of cytokines, which in turn promotes the presentation of tumor-associated antigens (TAAs) on antigen-presenting cells (APCs), which leads to the aggregation of anti-tumor-activated immune cells, such as effector T cells, Treg cells, dendritic cells (DCs) and so on [6]. Therefore, radiotherapy can synergistically collaborate with the ICIs by bolstering the immune system. Research indicates that radiation therapy can upregulate the expression of PD-L1 on tumor cells [7], thereby enhancing the therapeutic efficacy of certain ICIs.

Nevertheless, ICIs and radiotherapy can elicit adverse effects on various organ systems. While activating anti-tumor immunity, PD-1 inhibitors may also cause the immune system to attack normal tissues. Adverse events are caused mainly through the abnormal activation of T cells and the release of inflammatory factors. Because the radiation cannot be completely confined to the tumor tissue during treatment, the surrounding normal tissue may be affected, causing adverse reactions, such as cough, upper respiratory tract infection, and radiation pneumonia, which are common adverse reactions of the respiratory system. Although many prospective clinical trials have been initiated in recent years to evaluate the safety and efficacy of combined RT + ICIs regimens, most of these are still ongoing and safety results are awaited.This systematic review and meta-analysis focus on the respiratory system, analyzing respiratory adverse effects in patients receiving a combination of the ICIs and radiotherapy. The objective is to assess the risk associated with these respiratory complications and provide insights for clinical interventions.

## Methods

This systematic review and meta-analysis was reported in accordance with the Preferred Reporting Items for Systematic Reviews and Meta-Analyses (PRISMA) Statement and was registered at the International Prospective Register of Systematic Reviews (PROSPERO, CRD42023461008).

### Search strategy

We searched the literature on ICIs combined with radiotherapy for the treatment of cancers from the following databases: PubMed, Embase, Cochrane Library, and Clinicaltrials (https://clinicaltrials.gov/) up to Jul 2023. We used the following combined text and MeSH terms: “Radiotherapy” ,“Immune Checkpoint Inhibitors”, “clinical trials” as a topic, and we connected them with “and” .

### Selection criteria

We included literature according to the following criteria: (1) Study type: randomized controlled trial (RCT), non-randomized controlled trial, prospective clinical trials, retrospective cohort studies, prospective case-control studies, and single-arm trials. (2) Histologically confirmed cancers. (3) It contains information on ICIs, radiotherapy, and adverse respiratory effects. Some exclusion criteria also include (1) In vitro or animal experiments. (2) The exact data in the article cannot be extracted. (3) Reviews, letters, unfinished studies, or duplicate reports.

### Data extraction

Two independent reviewers read the study titles and abstracts, screened the literature according to the inclusion and exclusion criteria mentioned above, and independently extracted the relevant data of the included articles, mainly including the name of the first author, year of publication, study design, study phase, type of ICIs, histology, treatment, radiotherapy dose and fraction, sample size and the number of patients with adverse respiratory effects(cough, pneumonitis, upper respiratory tract infection). The third reviewer decides on disagreements in the evaluation.

### Quality assessment

The quality assessment of Randomized Controlled Trials (RCTs) was conducted using the Cochrane risk of bias tool. Quality assessment was performed using the MINORS scale [8] for single-arm and non-randomized controlled studies.

### Statistical analysis

We used the I^2^ statistics to evaluate the heterogeneity. The random effect model was used when the I2 value was over 50%. On the contrary, the fixed effect model was used. Subgroup analyses were conducted to probe the source of the heterogeneity. Publication bias analysis was performed using a funnel plot, Egger’s test, and Begg’s test. All the analyses above were conducted by Rstudio. *P* < 0.05 indicated a statistically significant difference.

## Results

### Studies selection and characteristics

**Fig. 1:**
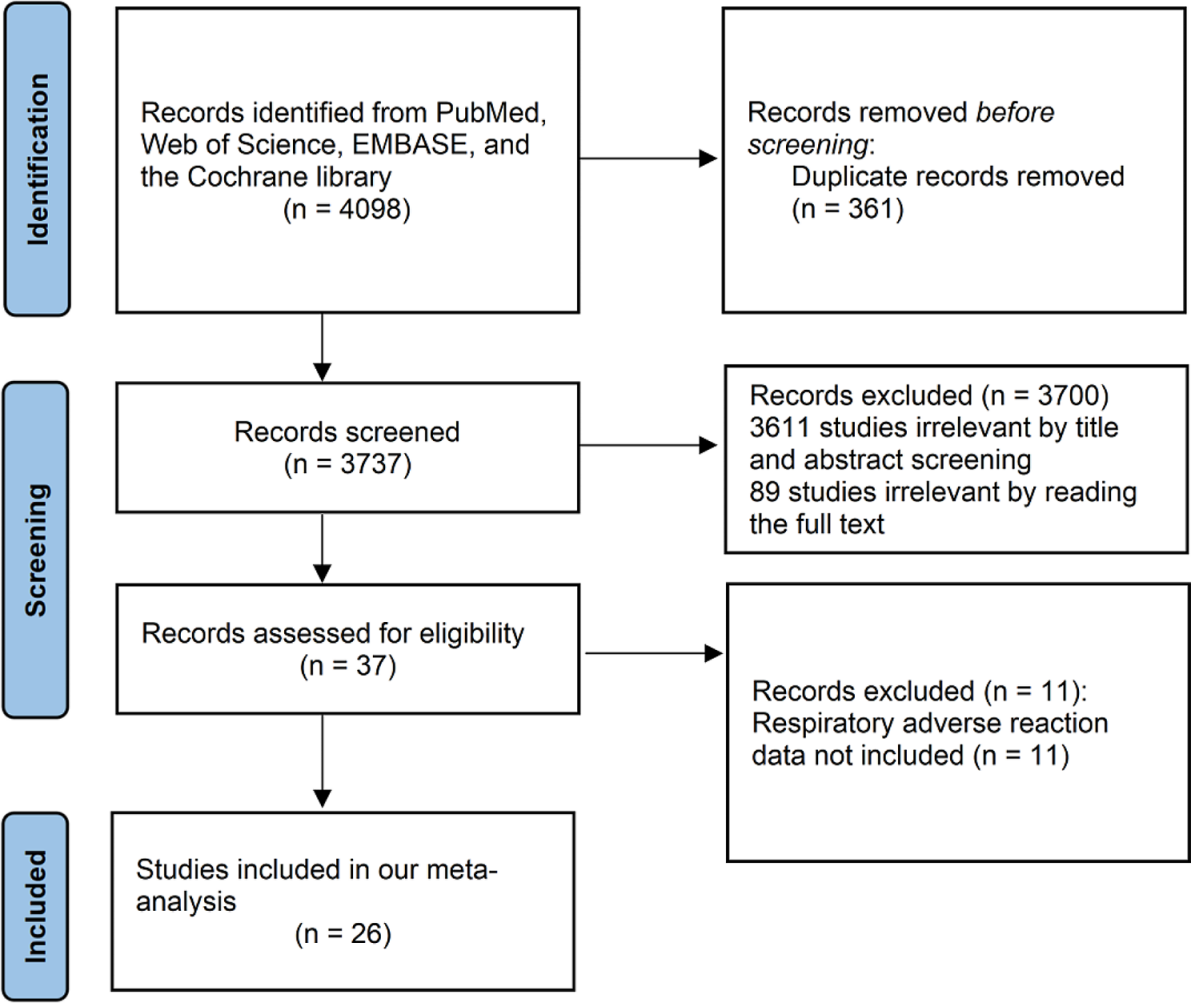
Study methodology flowchart. All the steps of this study, from the start to the end are described in the given diagram

After the primary search, we screened a total of 4098 articles in four databases, and after excluding 361 duplicates, two independent reviewers screened the titles and abstracts of the remaining 3737 articles and excluded 3611 irrelevant articles. After reading the full text of the remaining 126 articles, 89 articles were excluded, and 26 of the remaining 37 articles contained data related to respiratory adverse effects, these 26 articles and 2670 patients were included in our meta-analysis [9–34]. The PRISMA flowchart is shown in Fig. 1.

In the selection of ICIs, a total of 15 studies opted for the solitary application of PD-1, while six studies chose to exclusively employ PD-L1. Additionally, four studies opted for the combined use of multiple ICIs, and one study consisted of two separate cohorts, one utilizing PD-L1 monotherapy and the other employing combination therapy. Regarding the choice of radiotherapy modality, seven studies utilized Stereotactic Body Radiation Therapy (SBRT), 12 studies employed alternative radiotherapy modalities, and seven studies did not explicitly specify the RT method. Further details on the baseline characteristics of the clinical trials included in this meta-analysis can be found in Table 1.

**Table 1:** Baseline characteristics of the eligible studies

First Author	Year	Study design	Phase	Histology	Type of ICIs	Treatment in the Treatment Group	Radiotherapy modalities	Radiotherapy dose and fraction	Sample size	Cough (grades 1–5, *n*)	Cough (grades 3–5, *n*)	Pneu-monia (grades 1–5, *n*)	Pneum-onia (grades 3–5, *n*)	Upper respiratory tract infection (grades 1–5, *n*)	Upper respiratory tract infection (grades 3–5, *n*)	Dyspnoea (grades 1–5, *n*)	Dyspnoea (grades 3–5, *n*)
Joe [9]	2023	Open-label, RCT	II	Early-Stage Non-Small Cell Lung Cancer	PD-1	Nivolumab + RT	SBRT	50 Gy/4Fx or 70 Gy/10Fx	66	NA	NA	1	1	NA	NA	1	1
Zhu [10]	2023	Single-arm	II	Locally Advanced Oesophageal Squamous Cell Carcinoma	PD-1	Toripalimab + Paclitaxel + Cisplatin + RT	IMRT	50.4 Gy/28Fx	42	29	1	28	2	NA	NA	NA	NA
Omuro [11]	2023	Open-label, RCT	III	MGMT-unmethylated Glioblastoma	PD-1	Nivolumab + RT	Focal RT	60 Gy/30Fx	278	2	0	2	1	NA	NA	1	1
Wise-Draper [12]	2022	Open-label, Nonrandomized, Double-arm	II	Resectable Local–Regionally Advanced Head and Neck Squamous Cell Carcinoma	PD-1	Pembrolizumab + Surgery ± Cisplatin + RT	IMRT	60–66 Gy/30-33Fx	92	8	0	2	2	NA	NA	2	1
Li [13]	2022	Open-label, Single-arm	II	Unresectable Hepatocellular Carcinoma	PD-1	Camrelizumab + RT	SBRT	30–50 Gy/10Fx	21	NA	NA	1	0	NA	NA	NA	NA
Kwan [14]	2022	Single-arm	II	Metastatic Castration-Resistant Prostate Cancer	PD-L1	Avelumab + RT	SBRT	20 Gy/1Fx	31	1	0	1	1	NA	NA	NA	NA
Chao [15]	2022	Single-arm	II	Metastatic Gastric, Gastroesophageal Junction, or Esophageal either Squamous Cell or Adenocarcinoma	PD-1	Pembrolizumab + RT	NA	30 Gy/10Fx	14	NA	NA	2	0	NA	NA	NA	NA
Lim [16]	2022	Single-blind, RCT	III	Supratentorial Glioblastoma	PD-1	Nivolumab + Temozolomide + RT	NA	60 Gy/NA	354	58	0	17	17	20	0	29	4
Kim [17]	2022	Open-label, RCT	II	Unresectable, Recurrent, or Stage IV Merkel Cell Carcinoma	PD-1 + CTLA-4	Nivolumab + Ipilimumab + RT	SBRT	24 Gy/3Fx	24	6	0	3	1	1	0	5	0
Zhou [18]	2022	Double-blind, RCT	III	Unresectable Stage III Non-Small Cell Lung Cancer	PD-L1	Sugemalimab(after cCRT/sCRT) + Platinum-based Chemotherapy + RT	NA	54–66 Gy/NA	255	NA	NA	57	13	NA	NA	NA	NA
Tai [19]	2021	Open-label, Single-arm	II	Hepatocellular Carcinoma with Child-Pugh A cirrhosis	PD-1	Nivolumab + Y90-radioembolisation	SIRT	-	36	1	0	1	0	NA	NA	NA	NA
Peters [20]	2021	Single-arm	II	Locally Advanced Stage IIIA-B Non-Small Cell Lung Cancer	PD-1	Nivolumab + Cisplatin/Carboplatin + Vinorelbine/Etoposide/Pemetrexed + RT	NA	66 Gy/33Fx	79	31	0	34	9	7	0	26	1
Ni [21]	2021	Open-label, Single-arm	II	Metastatic Non-Small Cell Lung Cancer	PD-1	Sintilimab + GM-CSF + RT	SBRT	24 Gy/3Fx	20	1	0	NA	NA	NA	NA	NA	NA
Segal [22]	2021	Single-arm	II	Metastatic Colorectal Cancer	PD-L1 + CTLA-4	Nivolumab + Tremelimumab + RT	EBRT	20–70 Gy/3-30Fx	24	1	0	NA	NA	NA	NA	1	0
Mayadev [23]	2020	Double-blind, RCT	III	Locally Advanced Cervical Cancer	PD-L1	Durvalumab + Cisplatin/Carboplatin + RT	EBRT + BCRT	45 Gy/25Fx + 27.5–30 Gy or 35–40 Gy/NA	385	NA	NA	2	2	24	0	1	1
Jabbour [24]	2020	Nonrandomized Controlled Trial	I	Unresectable Stage IIIA or IIIB Non-Small Cell Lung Cancer	PD-1	Pembrolizumab(before, after and whole CRT) + Carboplatin + Paclitaxel + RT	IMRT/VMAT/Proton	60 Gy/30Fx	21	16	0	7	2	NA	NA	16	2
Elbers [25]	2020	Single-arm, Feasibility Trial	I	Advanced-Stage Head and Neck Squamous Cell Carcinoma of the Oral Cavity, Oropharynx, Hypopharynx or Larynx	PD-L1	Aavelumab + Cetuximab + RT	VMAT	Elective irradiation 46 Gy/23Fx then sequential boost 24 Gy/12Fx to primary tumor or 54.25 Gy/35Fx with SIB 70 Gy to primary tumor	10	NA	NA	3	2	NA	NA	NA	NA
Barroso-Sousa [26]	2020	Single-arm	II	Hormone Receptor-Positive Metastatic Breast Cancer	PD-1	Pembrolizumab + RT	NA	20 Gy/5Fx	8	1	0	NA	NA	1	0	2	0
Xie [27]	2020	Open-label, Nonrandomized, Two-cohort, Four-arm	I	Metastatic Pancreatic Ductal Adenocarcinoma	Cohort A: PD-L1 Cohort B: PD-L1 + CTLA-4	Cohort A: Durvalumab + RT Cohort B: Durvalumab + Tremelimumab + RT	SBRT	Cohort A1: 8 Gy/1Fx, Cohort A2: 5 Gy/5Fx, Cohort B1: 8 Gy/1Fx, Cohort B2: 5 Gy/5Fx	14, 11, 19, 21	1, 1, 3, 3	0, 0, 0, 0	0, 1, 2, 0	0, 1, 0, 0	1, 0, 0, 1	0, 0, 0, 0	4.2.6.7	1.0.1.1
Yu [28]	2019	Double-blind, RCT	III	Locoregionally Advanced Head and Neck Squamous Cell Carcinoma	PD-L1	Avelumab + Cisplatin + RT	IMRT	70 Gy/35Fx	345	74	0	61	25	NA	NA	40	7
Gerber [29]	2017	Double-blind, RCT	III	Locally Advanced Non-Small Cell Lung Cancer	PD-1	Nivolumab + Cisplatin + Etoposide + Thoracic RT	3DCRT/IMRT	60 Gy/30Fx	3	3	0	2	1	NA	NA	1	0
NCT(03102242) [30]	NA	Single-arm	II	Unresectable Stage III Non-Small Cell Lung Cancer	PD-L1	Atezolizumab + Carboplatin + Paclitaxel + RT	NA	60 Gy/NA	64	29	0	15	5	11	1	34	1
NCT(04081688) [31]	NA	Single-arm	I	Metastatic Non-Small Cell Lung Cancer	PD-L1 + CD27	Atezolizumab + Varlilumab + RT	SBRT	NA/NA	15	2	0	3	3	NA	NA	6	4
NCT(03421652) [32]	NA	Single-arm	II	Urothelial Bladder Cancer	PD-1	Nivolumab + RT	NA	64 Gy/32-35fX	20	3	0	NA	NA	NA	NA	3	NA
NCT(03040999) [33]	NA	Double-blind, RCT	III	Locally Advanced Head and Neck Squamous Cell Carcinoma	PD-1	Pembrolizumab + Cisplatin + RT	AFX/SFX	70 Gy/35Fx	398	65	0	66	43	NA	NA	22	2
NCT(02659540) [34]	NA	Open-label, Double-arm	I	Unresectable Stage IV Melanoma	PD-1 + CTLA-4	Divolumab + Ipilimumab + RT	Arm1: Conventional, Arm2: Hypofractionated	30 Gy/10Fx, 27 Gy/3Fx	10, 10	2, 4	0, 1	1, 2	0, 0	2, NA	1, NA	4.3	2.2

### Risk of Respiratory adverse effects in patients treated with immune checkpoint inhibitors in combination with radiotherapy

**Fig. 2:**
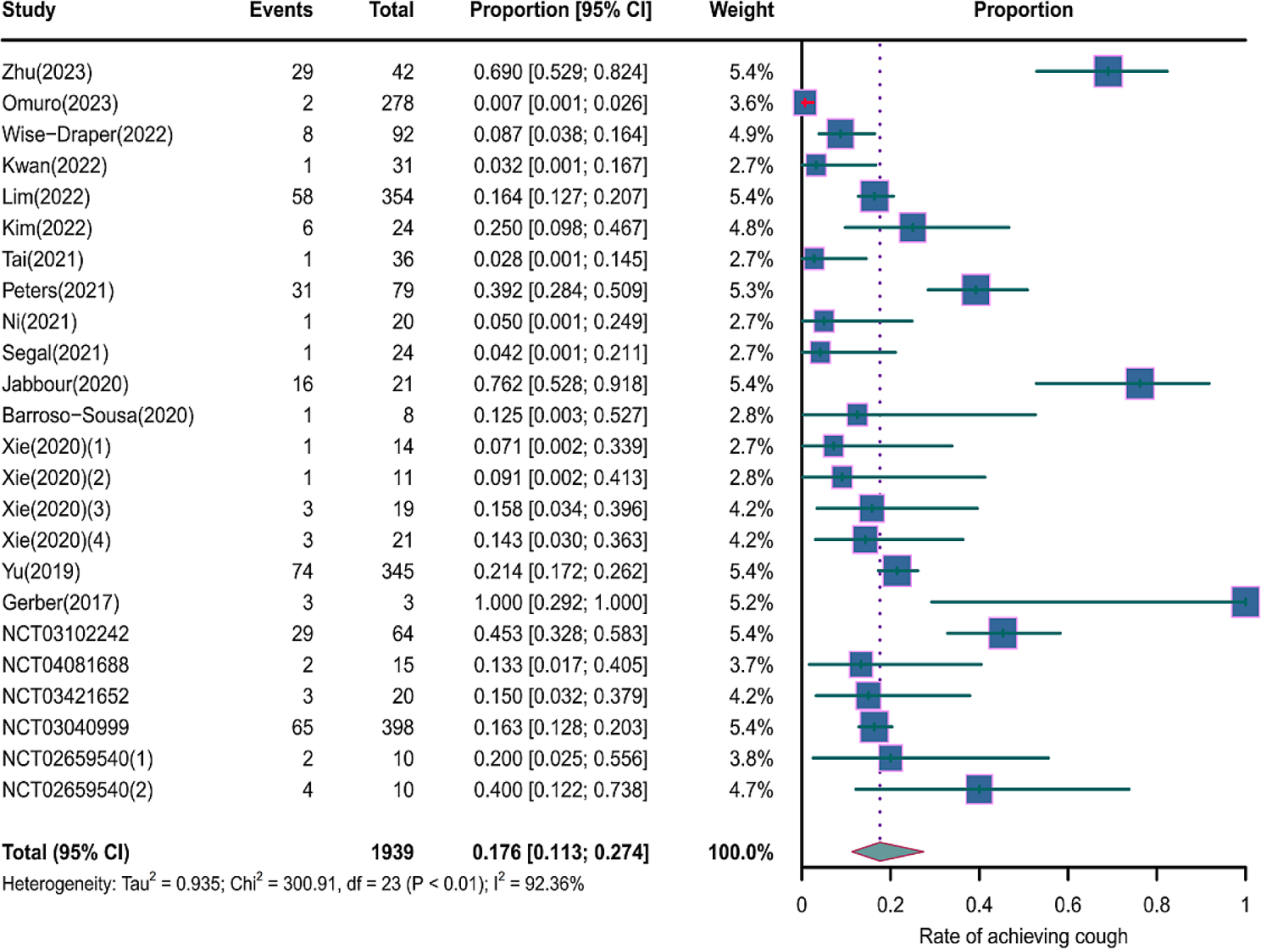
Forest plot of incidence rate of grades 1–5 cough in Patients Treated with Immune Checkpoint Inhibitors in Combination with Radiotherapy

**Fig. 3:**
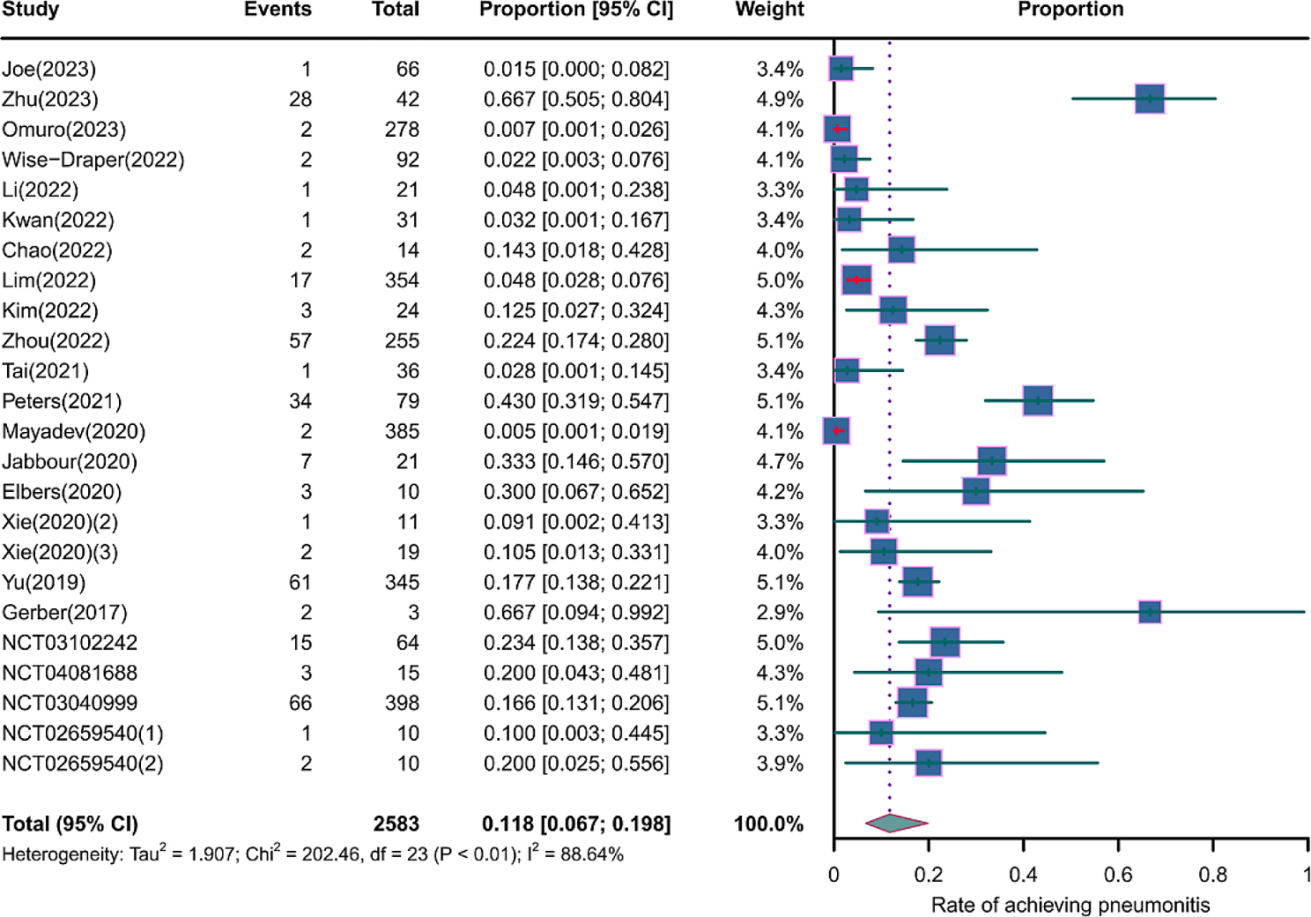
Forest plot of incidence rate of grades 1–5 pneumonitis in Patients Treated with Immune Checkpoint Inhibitors in Combination with Radiotherapy

**Fig. 4:**
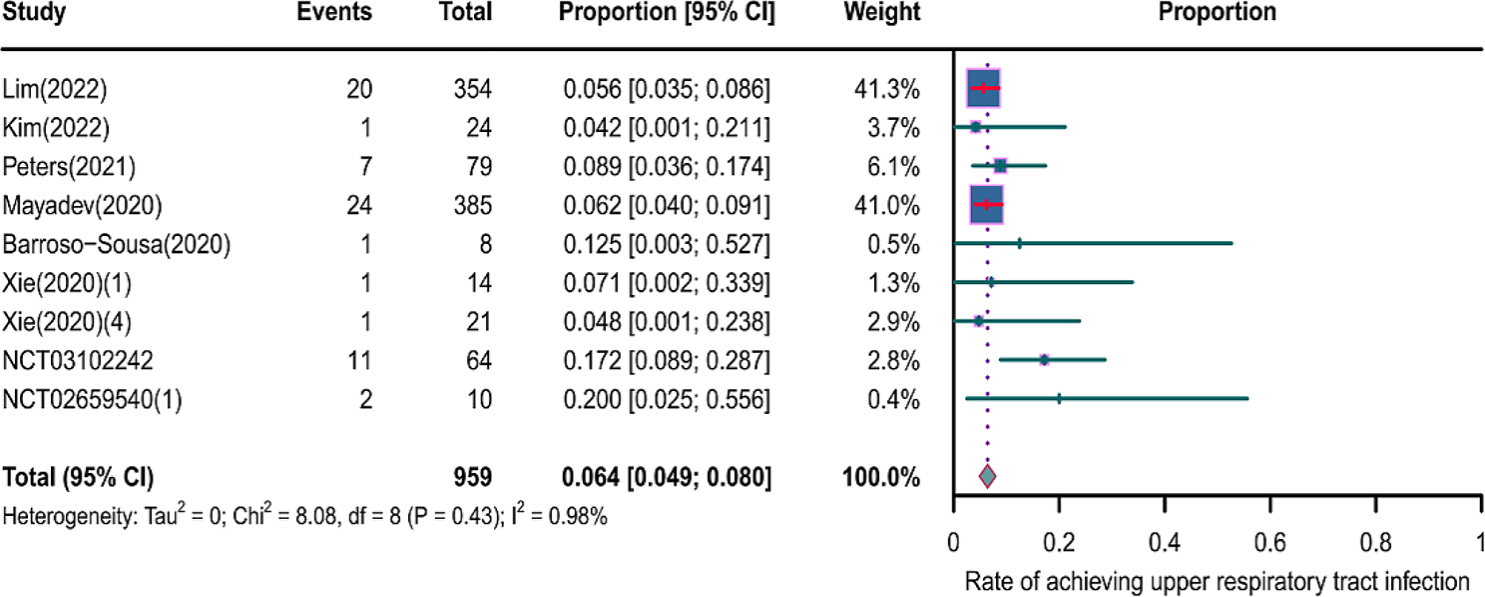
Forest plot of incidence rate of grades 1–5 upper respiratory tract infection in Patients Treated with Immune Checkpoint Inhibitors in Combination with Radiotherapy

**Fig. 5:**
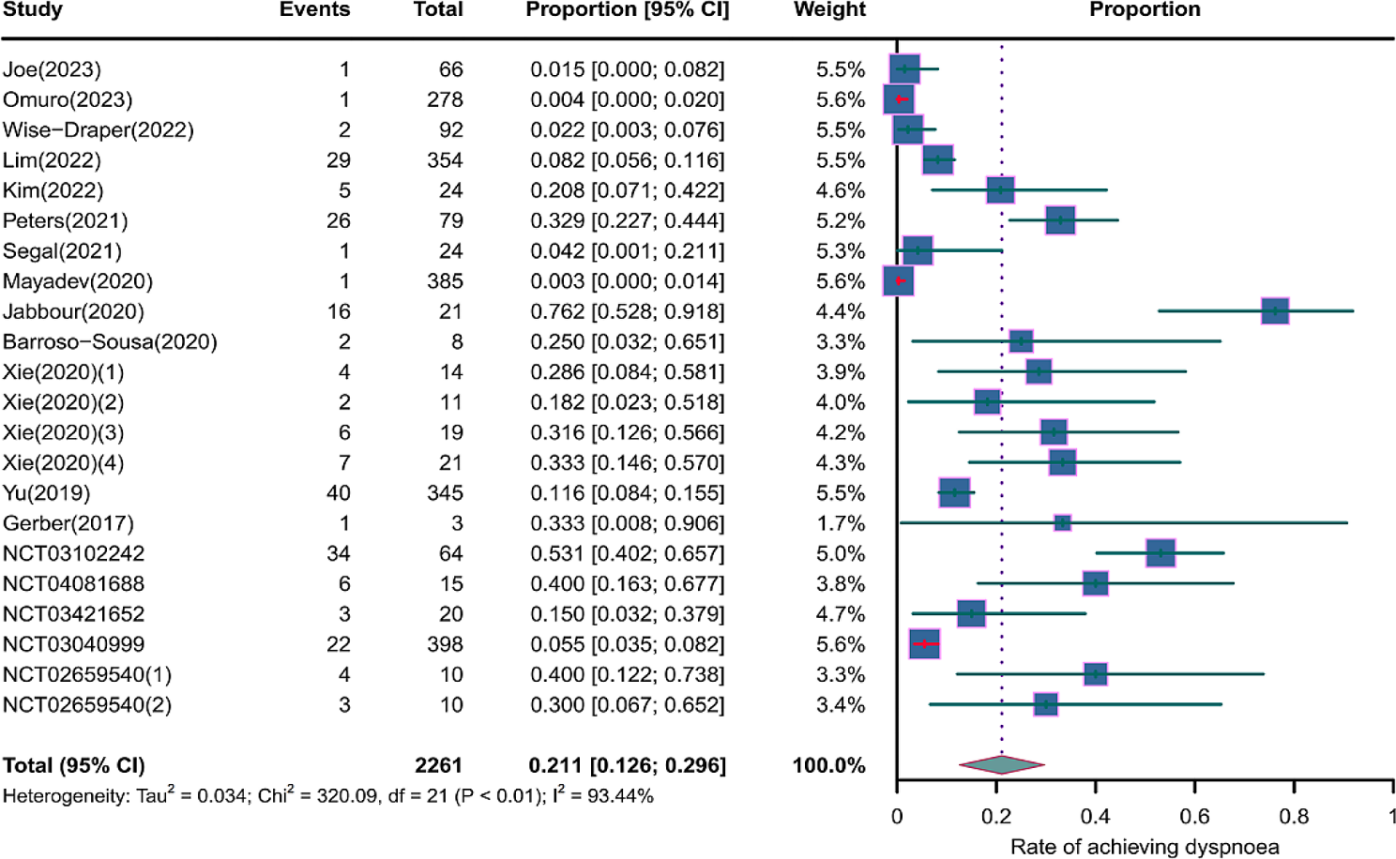
Forest plot of incidence rate of grades 1–5 dyspnea in Patients Treated with Immune Checkpoint Inhibitors in Combination with Radiotherapy

We evaluated the incidence rates of adverse respiratory events, including cough, pneumonia, upper respiratory tract infections, and others. Specifically, the incidence rates for different adverse events were as follows: the highest incidence rate of adverse events was for grades 1–5 cough, with an incidence rate of 0.176 (95%CI: 0.113–0.274, I^2^ = 92.36%); the incidence rate of grades 1–5 pneumonitis was 0.118 (95%CI: 0.067–0.198, I^2^ = 88.64%); the incidence rate of grades 1–5 upper respiratory tract infection was 0.064 (95%CI: 0.049–0.080, I^2^ = 0.98%)(Figs. 2, 3 and 4). The incidence rate of severe adverse events was relatively similar: the incidence rate of grades 3–5 cough was 0.050 (95%CI: 0.012–0.204, I^2^ = 8.90%); the incidence rate of grades 3–5 pneumonitis was 0.052 (95%CI: 0.031–0.078, I^2^ = 83.86%); the incidence rate of grades 3–5 upper respiratory tract infection was 0.040 (95%CI: 0.007–0.249, I^2^ = 45.31%)(Supplementary Figs. 1–3). In addition, we also assessed the incidence rate of dyspnea. The incidence rate of grades 1–5 dyspnea was 0.211 (95%CI: 0.126–0.296, I^2^ = 93.44%)(Fig. 5), while the incidence rate of grades 3–5 dyspnea was 0.029 (95%CI: 0.014–0.061, I^2^ = 75.34%)(Supplementary Fig. 4).

### Risk of respiratory adverse effects in different Immune checkpoint inhibitors therapies

**Fig. 6:**
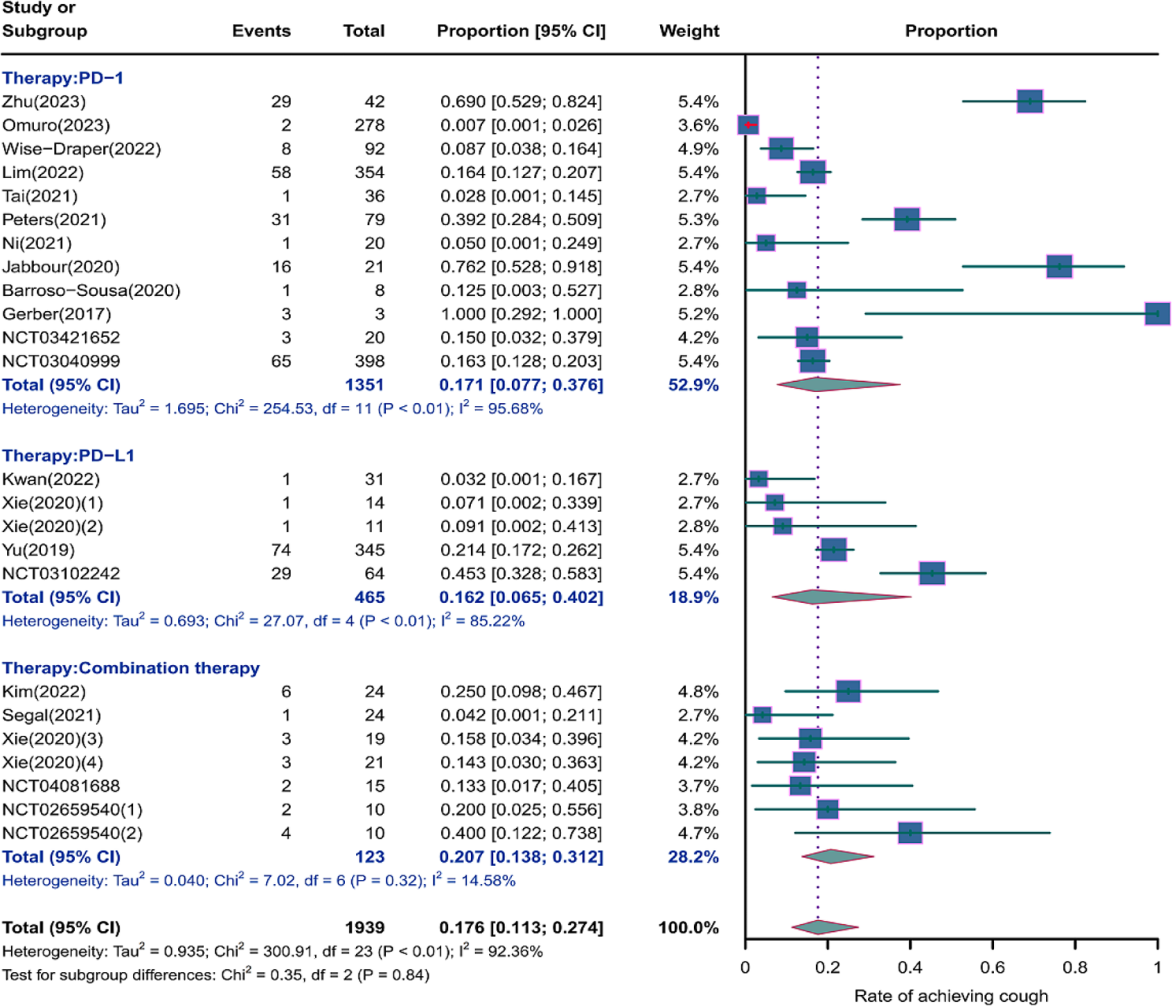
Forest plot of incidence rate of grades 1–5 cough for subgroup analysis by different immune checkpoint inhibitors therapies

**Fig. 7:**
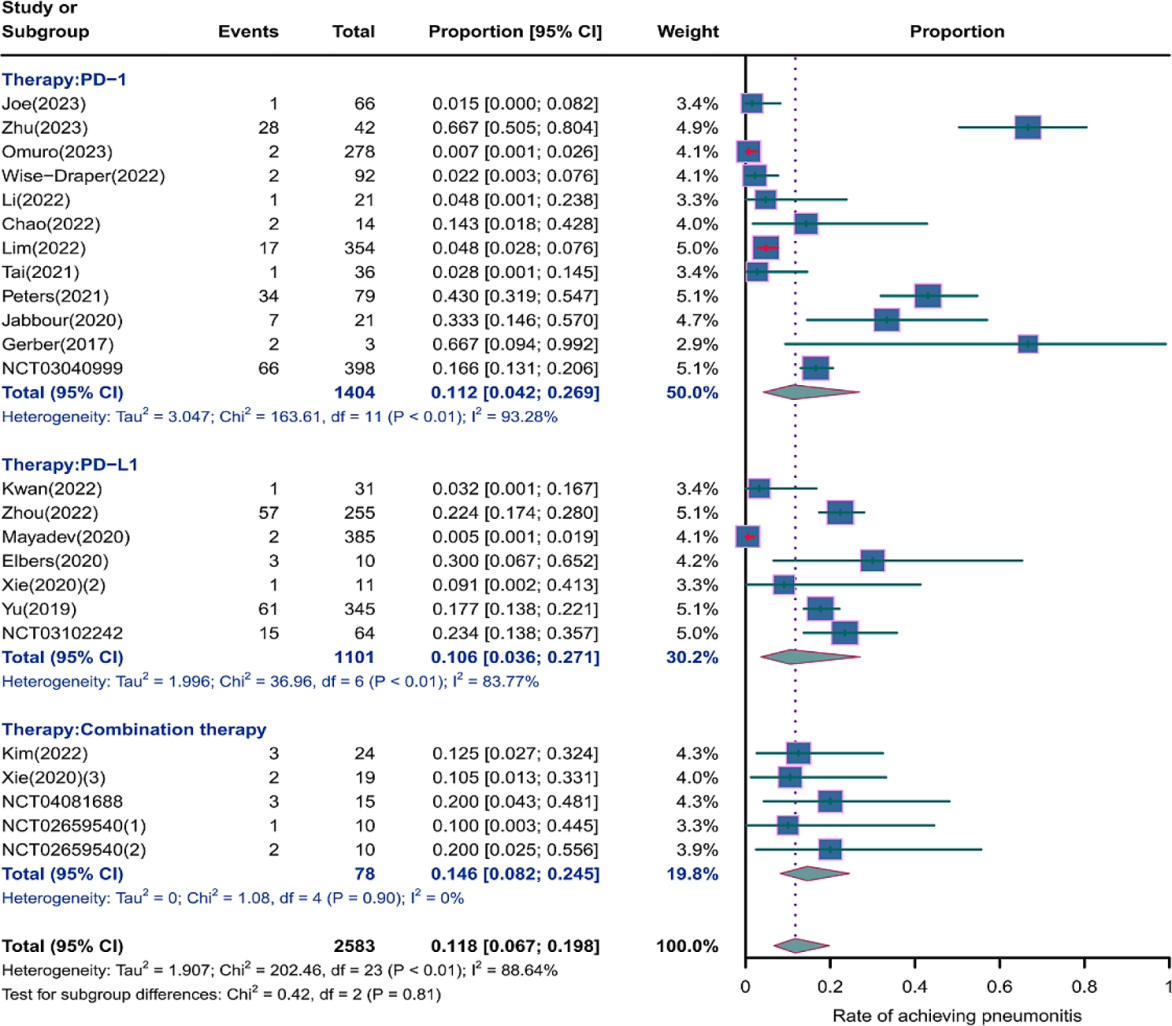
Forest plot of incidence rate of grades 1–5 pneumonitis for subgroup analysis by different immune checkpoint inhibitors therapies

**Fig. 8:**
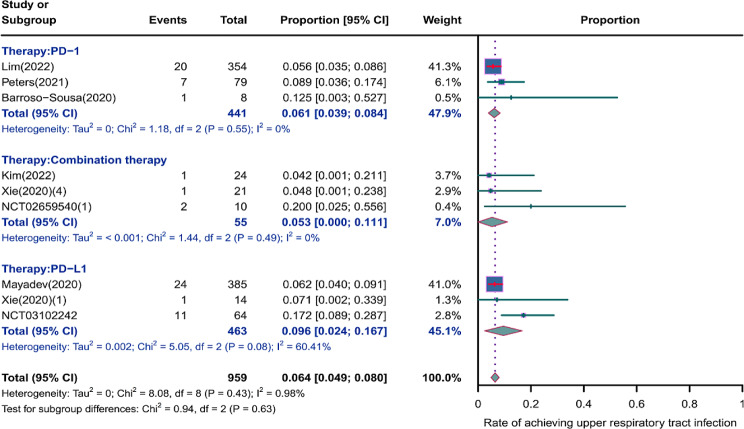
Forest plot of incidence rate of grades 1–5 upper respiratory tract infection for subgroup analysis by different immune checkpoint inhibitors therapies

As shown in the Figs. 6, 7 and 8, we conducted subgroup analyses on PD-1 inhibitor, PD-L1 inhibitor, and combination therapy respectively. We found that the incidence rate of respiratory adverse reactions(cough, pneumonitis, upper respiratory tract infection) in patients receiving combination therapy was higher than that in patients receiving PD-1 or PD-L1 inhibitor monotherapy. Regarding the comparison between PD-1 and PD-L1 inhibitor, except for a similar incidence rate of severe pneumonia, the incidence rate of other respiratory adverse reactions was higher with PD-1 inhibitor treatment(Supplementary Fig. 5).

The incidence rates for adverse events of applied combination therapy were as follows: the incidence rate of grades 1–5 cough was 0.207 (95%CI: 0.138–0.312, I^2^ = 14.58%); the incidence rate of grades 1–5 pneumonitis was 0.146 (95%CI: 0.082–0.245, I^2^ = 0%); the incidence rate of grades 1–5 upper respiratory tract infection was 0.096 (95%CI: 0.024–0.167, I^2^ = 60.41%); grades 3–5 pneumonitis, 0.100 (95%CI: 0.005–0.295, I^2^ = 59.34%). The incidence rates for adverse events of applied PD-1 inhibitor therapy were as follows: the incidence rate of grades 1–5 cough was 0.171 (95%CI: 0.077–0.376, I^2^ = 95.68%); the incidence rate of grades 1–5 pneumonitis was 0.112 (95%CI: 0.042–0.296, I^2^ = 93.28%); the incidence rate of grades 1–5 upper respiratory tract infection was 0.061 (95%CI: 0.039–0.084, I^2^ = 0%); the incidence rate of grades 3–5 pneumonitis was 0.049 (95%CI: 0.021–0.088, I^2^ = 86.96%). The incidence rates for adverse events of applied PD-L1 inhibitor therapy were as follows: the incidence rate of grades 1–5 cough was 0.162 (95%CI: 0.065–0.402, I^2^ = 85.22%); the incidence rate of grades 1–5 pneumonitis was 0.106 (95%CI: 0.036–0.271, I^2^ = 83.77%); the incidence rate of grades 1–5 upper respiratory tract infection was 0.053 (95%CI: 0.000-0.111, I^2^ = 0%); the incidence rate of grades 3–5 pneumonitis was 0.050 (95%CI: 0.020–0.091, I^2^ = 84.23%).

### Risk of respiratory adverse effects in different radiotherapies

**Fig. 9:**
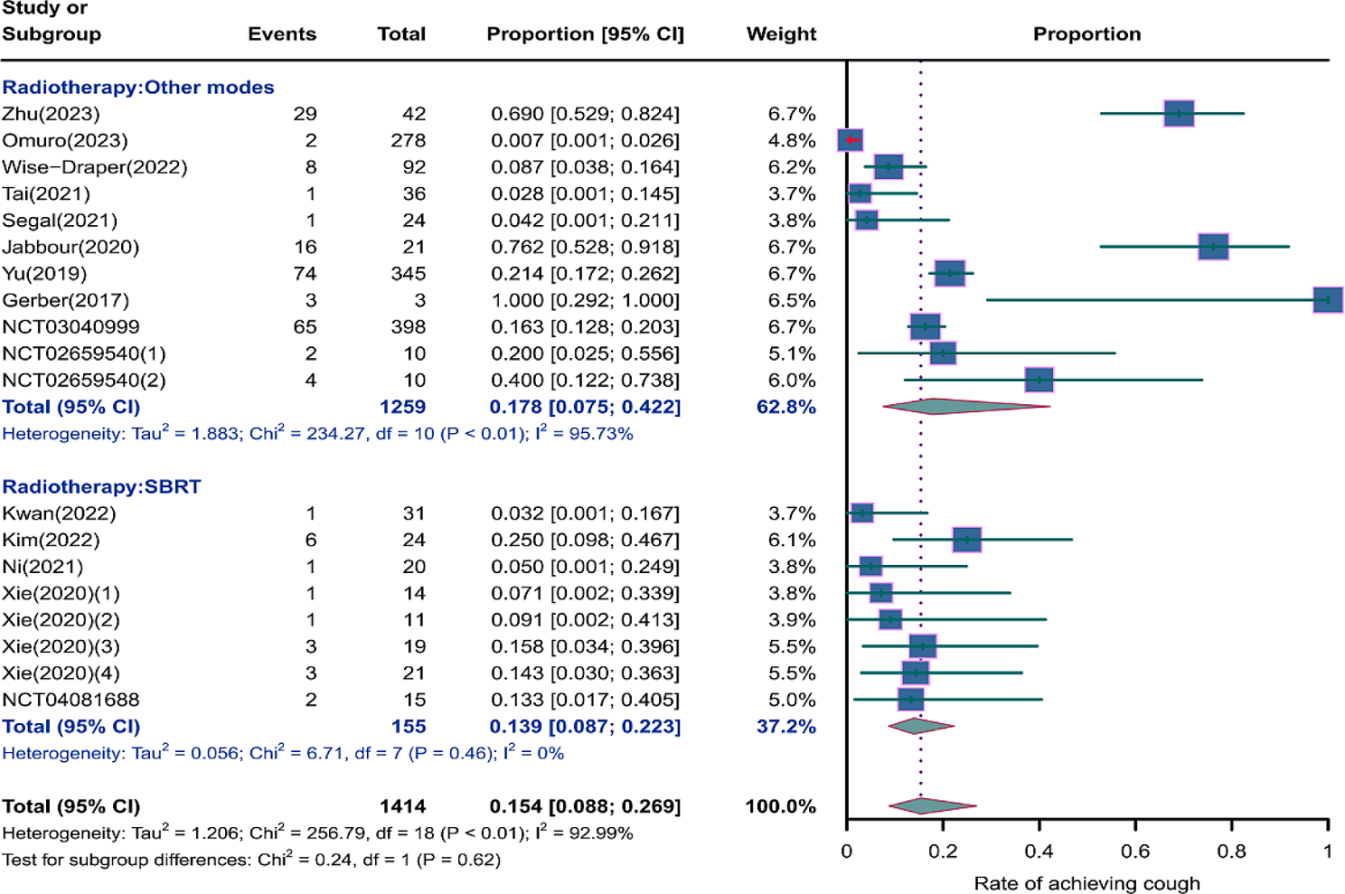
Forest plot of incidence rate of grades 1–5 cough infection for subgroup analysis by different radiotherapies

**Fig. 10:**
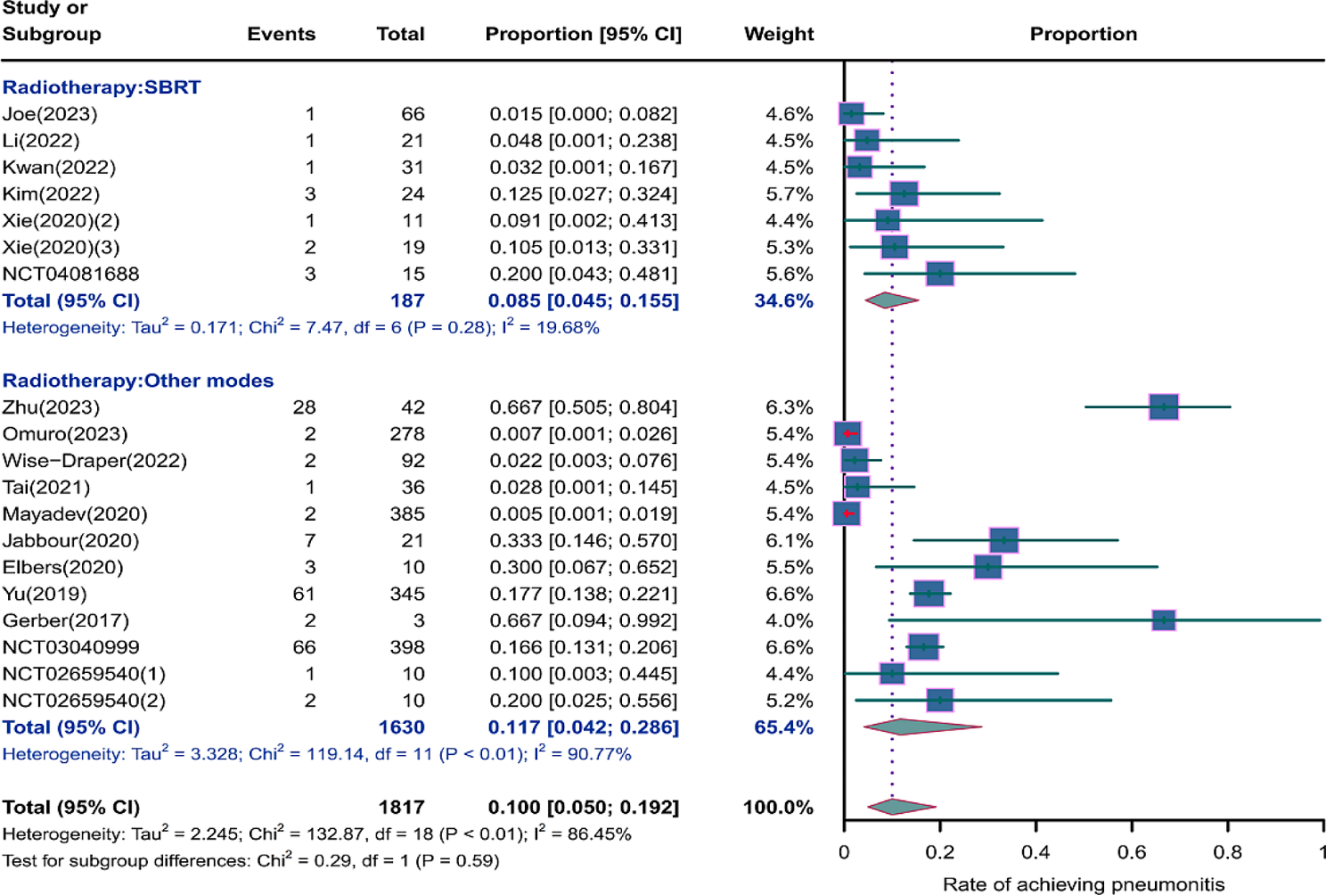
Forest plot of incidence rate of grades 1–5 pneumonitis for subgroup analysis by different radiotherapies

**Fig. 11:**
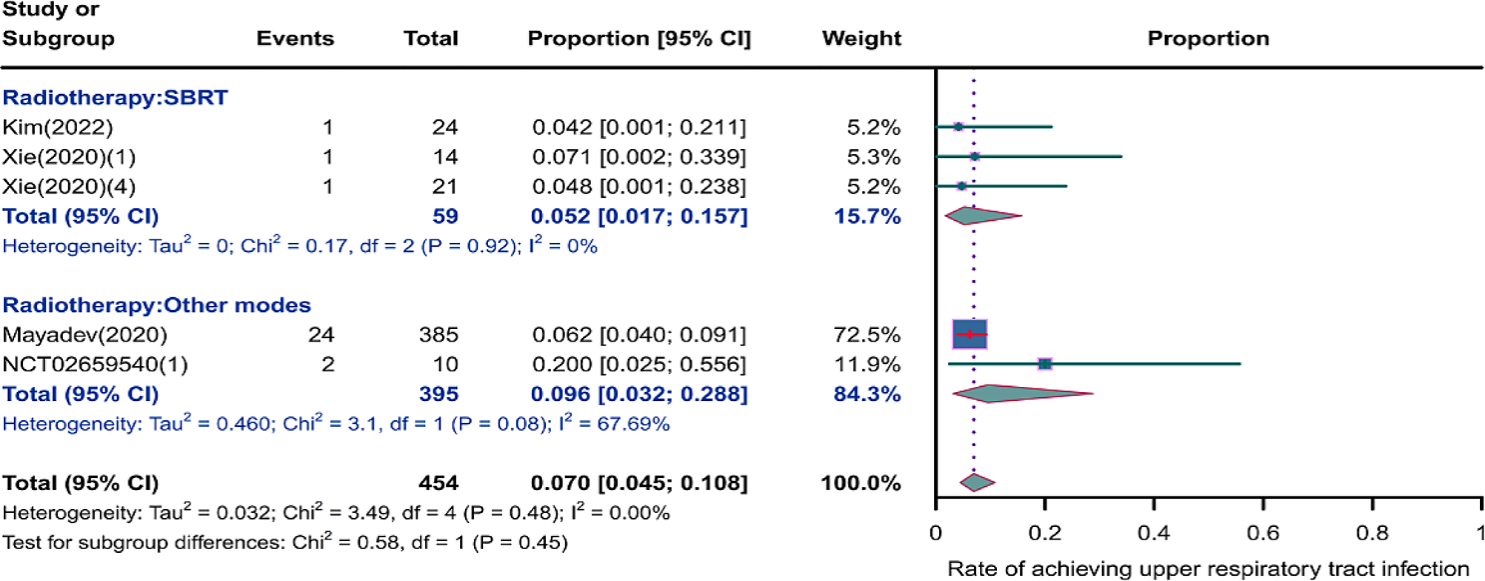
Forest plot of incidence rate of grades 1–5 upper respiratory tract infection for subgroup analysis by different radiotherapies

As shown in the Figs. 9, 10 and 11, compared to other radiotherapy modes, SBRT (stereotactic body radiotherapy) exhibited lower incidence rates for grades 1–5 cough (0.139, 95% CI: 0.0.087–0.223 vs. 0.178, 95% CI: 0.075–0.422), grades 1–5 pneumonitis (0.085, 95% CI: 0.045–0.155 vs. 0.117, 95% CI: 0.042–0.286), and grades 1–5 upper respiratory tract infection (0.052, 95% CI: 0.017–0.157 vs. 0.096, 95% CI: 0.032–0.288), but a higher incidence of grade 3–5 pneumonitis (0.061, 95% CI: 0.022–0.161 vs. 0.049, 95% CI: 0.019–0.120)(Supplementary Fig. 6).

### Risk of respiratory adverse effects in different tumor locations

**Fig. 12:**
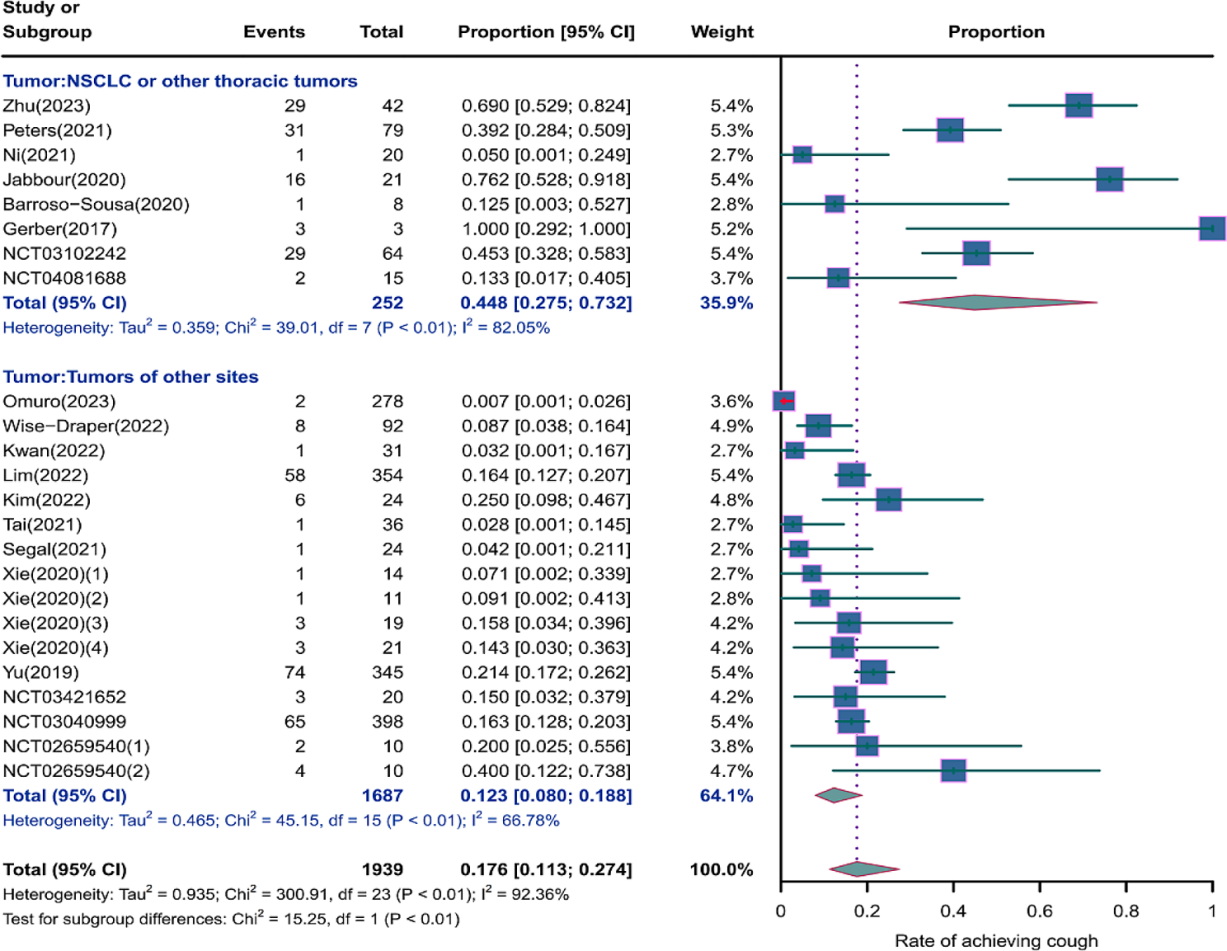
Forest plot of incidence rate of grades 1–5 cough for subgroup analysis by different tumor locations

**Fig. 13:**
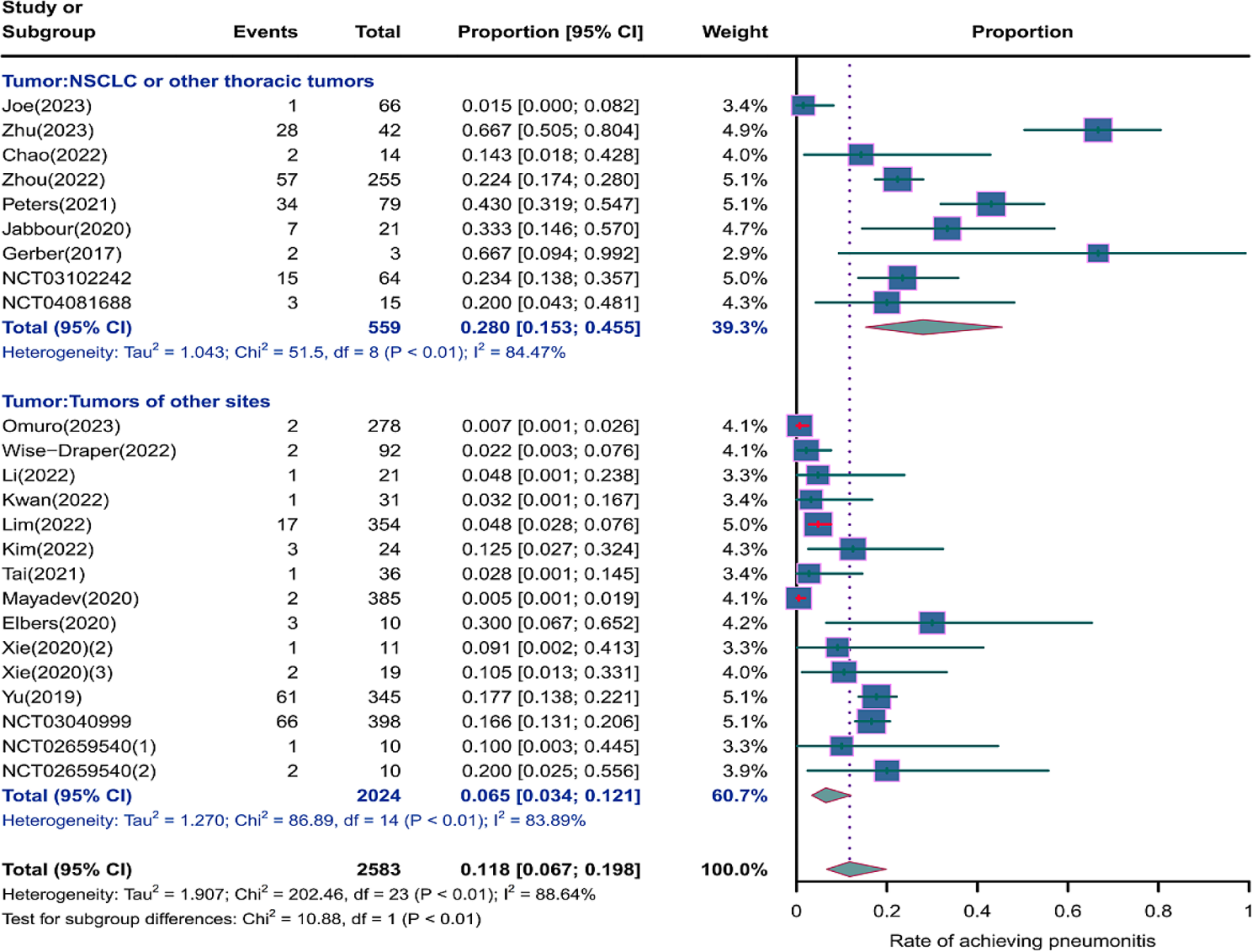
Forest plot of incidence rate of grades 1–5 pneumonitis for subgroup analysis by different tumor locations

**Fig. 14:**
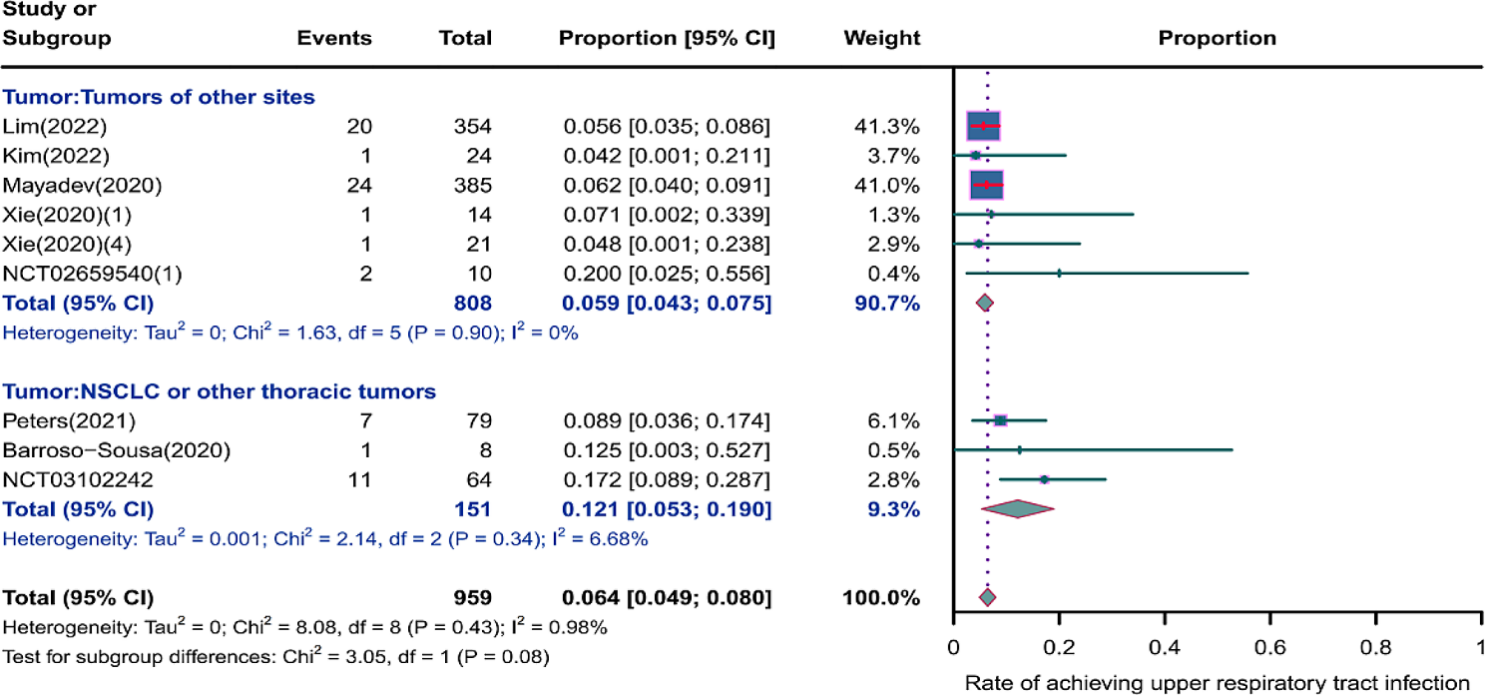
Forest plot of incidence rate of grades 1–5 upper respiratory tract infection for subgroup analysis by different tumor locations

As shown in the Figs. 12, 13 and 14, we performed a subgroup analysis of the tumor locations. The results showed that compared to tumors of other sites, NSCLC or other thoracic tumors had significantly higher incidence rates for grades 1–5 cough (0.488, 95% CI: 0.275–0.732 vs. 0.123, 95% CI: 0.080–0.188), grades 1–5 pneumonitis (0.280, 95% CI: 0.153–0.455 vs. 0.065, 95% CI: 0.034–0.121), grades 1–5 upper respiratory tract infection (0.121, 95% CI: 0.053–0.190 vs. 0.059, 95% CI: 0.043–0.075), and grades 3–5 pneumonitis (0.068, 95% CI: 0.038–0.105 vs. 0.041, 95% CI: 0.017–0.074)(Supplementary Fig. 7).

### Risk of respiratory adverse effects in different treatment orders

**Fig. 15:**
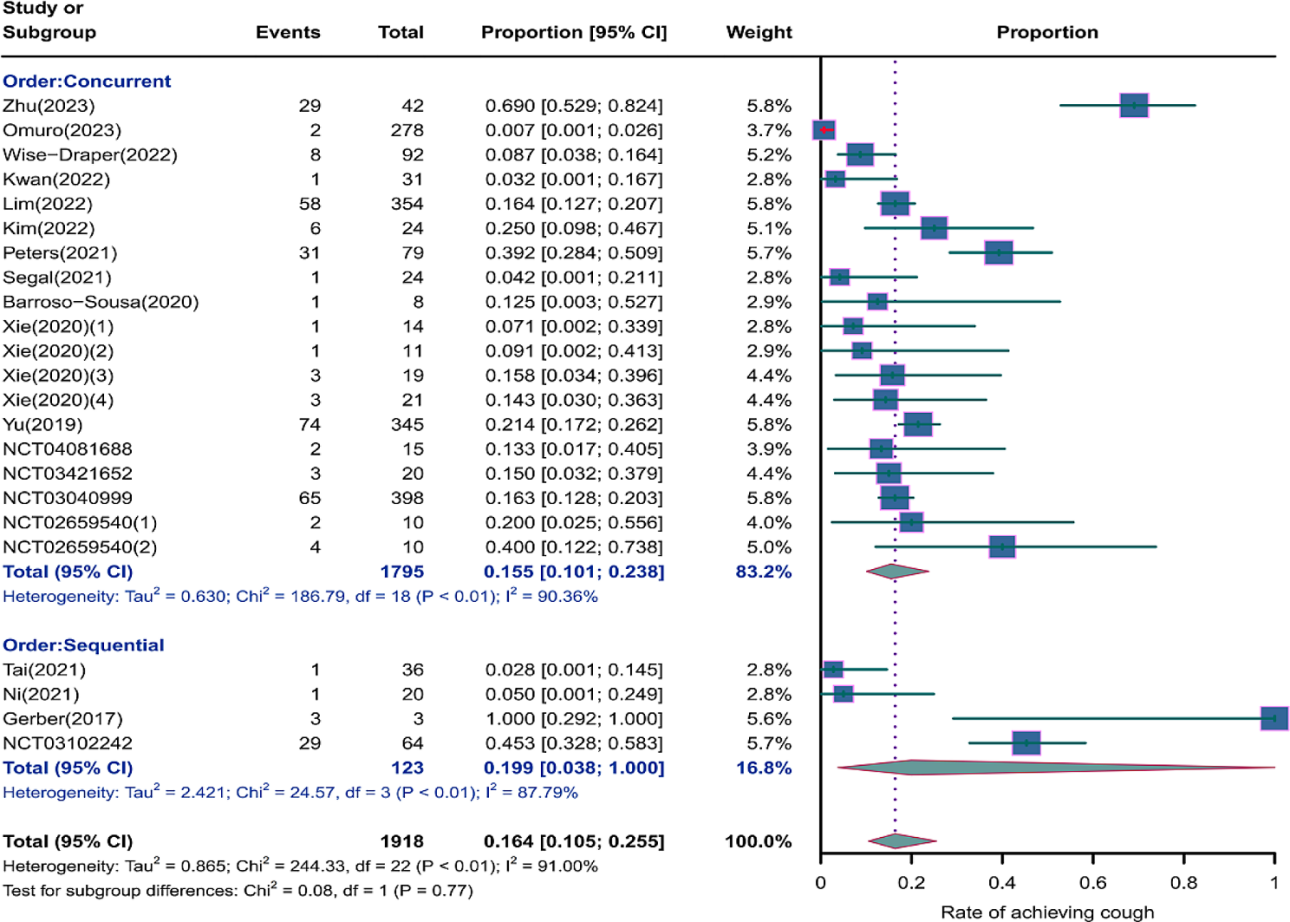
Forest plot of incidence rate of grades 1–5 cough for subgroup analysis by different treatment orders

**Fig. 16:**
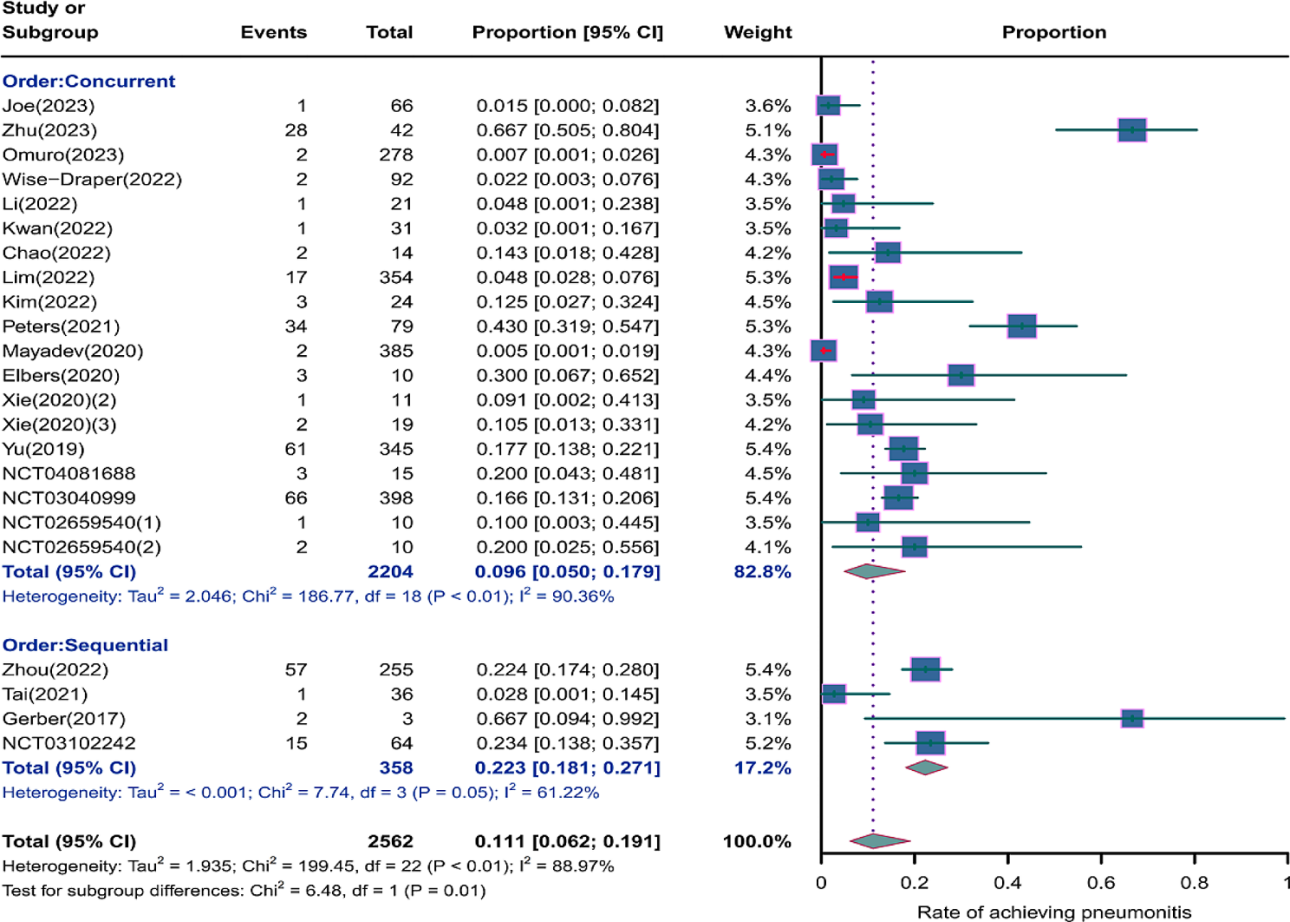
Forest plot of incidence rate of grades 1–5 pneumonitis for subgroup analysis by different treatment orders

**Fig. 17:**
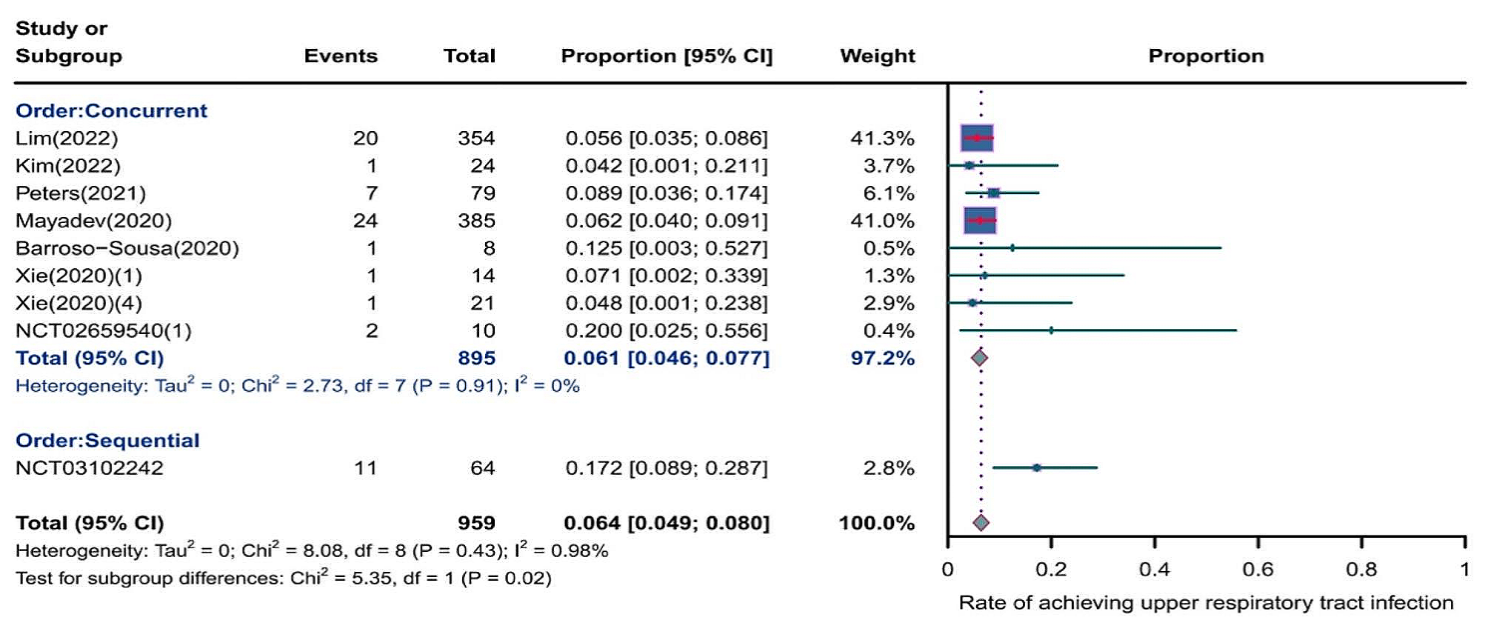
Forest plot of incidence rate of grades 1–5 upper respiratory tract infection for subgroup analysis by different treatment orders

As shown in the Figs. 15, 16 and 17, compared to sequential treatment, concurrent treatment exhibited lower incidence rates for grades 1–5 cough (0.155, 95% CI: 0.101–0.238 vs. 0.199, 95% CI: 0.038-1.000), grades 1–5 pneumonitis (0.096, 95% CI: 0.050–0.179 vs. 0.223, 95% CI: 0.181–0.271), and grades 3–5 pneumonitis (0.047, 95% CI: 0.024–0.078 vs. 0.058, 95% CI: 0.035–0.086)(Supplementary Fig. 8).

### Quality Assessment and Publication Bias

We used the Cochrane risk of bias tool to assess the quality of RCTs, and the results are shown in Supplementary Fig. 9. As shown in the figure, we used the MINORS scale to assess the quality of single-arm and non-randomized controlled studies (Supplementary Table 1). We conducted publication bias analysis using funnel plots, and except for Dyspnoea, which displayed a noticeably asymmetric funnel plot, the funnel plots for the other adverse effects appeared relatively symmetrical (Supplementary Figs. 10–15). Egger’s and Begg’s tests were also performed to recognize publication bias in this studyl (Supplementary Figs. 16–21). Except for Dyspnoea, which showed a significant result with Egger’s test (*p* < 0.0001), no substantial bias was observed for the other adverse effects. Sensitivity analyses were also performed to assess the stability of the included studies (Supplementary Figs. 22–33). After excluding one study at a time, no significant difference in the results was found from the initial analysis.

## Discussion

An increasing body of clinical evidence suggests that radiotherapy induces local and systemic immune responses that can synergize with ICIs to enhance the efficacy of these treatments, ultimately improving patient outcomes [35–38]. However, the mechanisms underlying the synergistic anti-tumor effects of radiotherapy and ICIs may also lead to overlapping toxicity profiles [39]. This inevitably exposes the patient to various adverse effects, including respiratory adverse effects [40]. Therefore, we conducted this meta-analysis and systematic review to assess the impact of various factors on the respiratory adverse effects produced by the application of ICIs in combination with radiotherapy for the treatment of patients with solid tumors.

Radiotherapy is administered by inducing DNA damage ultimately leading to tumor cell killing [36, 41–43]. The essence of RT-induced AE is DNA damage to normal tissues, and the incidence and severity are related to the anatomic site of irradiation, the dosage/grading strategy of the treatment, and the volume of irradiation [44, 45]. The primary way to reduce rt-induced AE is to reduce the irradiation volume at regular sites, allowing less dose to be delivered to normal tissues but maintaining the therapeutic dose at the tumor site [46]. Immune-related adverse effect (irAE) is essentially an attack on normal tissues by reactivated immune cells leading to the emergence of an inflammatory response, including multisystem toxicity of the respiratory system, the gastrointestinal tract, the endocrine, the neurological system, and the skin [47, 48]. Most immune-related adverse drug effect (irade) symptoms are mild, long-lasting, and do not disappear immediately after discontinuation of the drug. However, severe irades such as pneumonia and myocarditis can be life threatening [49]. Grade 4 irade requires permanent discontinuation of ICIs and immunosuppressive treatment with high-dose steroids [50].

For the synergistic mechanism of radiotherapy combined with ICIs, the main point is that radiotherapy can enhance the immune response by remodeling the tumor microenvironment [51]. Current studies suggest that on the one hand, radiotherapy can directly activate innate and adaptive immune cells with various effects on tumor growth and tumor cell death. Ionizing radiation can lead to exposure of immunogenic molecules to the cell surface by inducing immunogenic cancer cell injury and cell death; damage-associated molecular patterns (DAMPs) such as S100 protein and adenosine triphosphate (ATP) are released to activate innate and adaptive immune responses [52, 53]. On the other hand, radiation-induced tissue damage is capable of releasing pro-inflammatory cytokines and activating humoral immune responses, recruiting innate immune cells such as granulocytes and macrophages, and enhancing the uptake of tumor-derived antigens by the antigen-presenting cells, which in turn affects tumorigenesis [54, 55].

The cost of the efficacy of radiation therapy combined with immunotherapy is the concomitant increase in AE [56]. Current studies suggest that the toxicities of RT and ICIs do not overlap, nor do they overlap completely [35]. Mechanistically, for the respiratory system, the mucosal barrier of the lung blocks pathogens, whereas epithelial cells and alveolar macrophages recognize pathogens and mediate immune responses [57]. During radiotherapy, radiation treatment causes some degree of damage to normal tissues, which is a very complex and dynamic process involving a close link between inflammation and injury. At the same time, innate immune cells, including neutrophils, monocytes, and macrophages, are the first line of defense against infection and release highly toxic chemicals to kill pathogens [58–60]. This tissue damage may excessively induce an inflammatory response in some patients and may evolve into abnormal inflammation. This toxic effect is not limited to the lungs and may develop into systemic side effects [61].

Our findings concluded that the combination of multiple immunosuppressants poses a greater risk of adverse effects than treatment with a single agent in combination with radiotherapy. At the same time, the safety profile of PD-L1 inhibitors may be better than that of PD-1 inhibitors. Two classic prospective studies have evaluated the safety of immunoradiotherapy combinations. The first, KEYNOTE-001, was a prospective secondary analysis of 97 patients and found that the overall incidence of pulmonary toxicity was 63% in patients who had received chest radiotherapy prior to treatment with pembrolizumab (anti-PD1), compared with 40% in those who did not receive radiotherapy [62]. Moreover, the incidence of all grades of ICI-related pulmonary toxicity was significantly higher in patients who had received radiotherapy in combination with immunotherapy (13% vs. 1%, *p* = 0.046). However, there was no significant relationship between the receipt of radiotherapy and the incidence of high-grade pulmonary toxicity. The second study was THE PACIFIC trial, a prospective, randomized, double-blind, placebo-controlled phase III study [63, 64]. This study compared the safety of receiving radiotherapy alone and radiotherapy combined with immunotherapy. In 713 patients, all grades of pneumonia occurred more frequently in the combination therapy group than in the radiotherapy alone group (33.9% vs. 24.8%). However, in high-grade pneumonia, no significant difference was found between the combination therapy and radiotherapy alone groups (3.4% vs. 2.6%). These two studies suggest that the use of immune response after radiotherapy may predispose to adverse pulmonary effects. In the clinic, we need to pay attention to the interval between radiotherapy and immunotherapy to avoid the concurrent use of immunosuppressants in the acute phase after radiotherapy or in combination with radiation pneumonitis, which may lead to severe pulmonary adverse events. Two meta-analyses comparing the pulmonary safety of anti-PD-1 and anti-PD-L1 showed that anti-PD-1 monoclonal antibodies led to a higher incidence of pneumonia than anti-PD-L1 monoclonal antibodies [35]. Mechanistically, it is possible that anti-PD-1 monoclonal antibodies are more likely to induce pulmonary adverse events in combination with radiotherapy because PD-L1 is expressed on the surface of tumor cells, and radiotherapy toxicity is more likely to cause damage and abnormal inflammatory responses in normal tissues [65].

Our other results, suggesting that the concurrent treatment modality would cause fewer pulmonary adverse effects compared to sequential treatment, may be related to the mechanism of occurrence of combined toxicity mentioned above. The use of immunosuppressive agents before irradiation causes altered damage to the tumor microenvironment and does not cause an excessive immune response. Also, the dose and volume of irradiation must be an essential factor influencing the adverse events of radiation therapy, which suggests that it is reasonable that SBRT would result in less pulmonary toxicity. We suggest that 8–12 Gy/fraction is preferred when performing radiotherapy and may minimize adverse events while maintaining the anti-tumor immune response. Regarding tumors from other sites causing pulmonary toxicity during treatment, we consider the Abscopal Effect relevant [66, 67]. During primary tumor treatment, radiotherapy combined with immunotherapy induced a systemic immune response that triggered a systemic anti-tumor effect, and non-radiated sites also gained involvement. This is accompanied by a certain degree of normal tissue damage, which appears to trigger other sites. However, the emergence of this pulmonary toxicity cannot be accepted as a result of the action of immunosuppressive agents alone because ICIs are a multisystemic broad-spectrum adverse event, especially after radiotherapy-enhanced immune response [68].

The limitation of our study is that we did not collect enough information about the specific radiotherapy regimen of the patients and did not obtain detailed information about the dose of radiotherapy, area of irradiation, duration of treatment, and sequential intervals between immunotherapy treatments, which are important considerations. This resulted in an insufficiently detailed subgroup analysis and prevented the validation and investigation of possible mechanisms of toxicity of the existing combination therapy.

## Conclusion

In conclusion, this meta-analysis suggested that the respiratory adverse effects of ICIs combined with radiotherapy in the treatment of solid tumors can be affected by different ICIs drugs, different radiotherapies, different tumor locations, and different treatment orders. For clinical applications, we suggest that 8–12 Gy/fraction is preferred when performing radiotherapy and may minimize adverse events while maintaining the anti-tumor immune response. Further investigation is needed to confirm this observation.

## Electronic supplementary material

Below is the link to the electronic supplementary material.

Supplementary Material 1

Supplementary Material 2

## Data Availability

No datasets were generated or analysed during the current study.
