# Supplementary material for: Respiratory adverse effects in patients treated with immune checkpoint inhibitors in combination with radiotherapy: a systematic review and meta-analysis
Source: Radiat Oncol. 2024 Oct 1;19:134. doi: 10.1186/s13014-024-02489-4 (PMC11445955; doi:10.1186/s13014-024-02489-4)
Supplement: Supplementary file 1 — Supplementary Material 1 [file 13014_2024_2489_MOESM1_ESM.doc]

Supplementary Table 1. Methodological Index for Non-Randomized Studies (MINORS) score chart to assess the quality of single-arm studies.

| First Author | Q1 | Q2 | Q3 | Q4 | Q5 | Q6 | Q7 | Q8 | Q9 | Q10 | Q11 | Q12 | Total |
| --- | --- | --- | --- | --- | --- | --- | --- | --- | --- | --- | --- | --- | --- |
| Zhu | 2 | 2 | 2 | 2 | 0 | 2 | 2 | 1 | NA | NA | NA | NA | 13 |
| Li | 2 | 2 | 2 | 2 | 0 | 2 | 2 | 1 | NA | NA | NA | NA | 13 |
| Kwan | 2 | 2 | 2 | 2 | 0 | 2 | 2 | 0 | NA | NA | NA | NA | 12 |
| Chao | 2 | 0 | 2 | 2 | 0 | 2 | 2 | 0 | NA | NA | NA | NA | 10 |
| Tai | 2 | 2 | 2 | 2 | 0 | 2 | 2 | 1 | NA | NA | NA | NA | 13 |
| Peters | 2 | 2 | 2 | 2 | 0 | 2 | 2 | 1 | NA | NA | NA | NA | 13 |
| Ni | 2 | 1 | 2 | 2 | 0 | 0 | 2 | 1 | NA | NA | NA | NA | 10 |
| Segal | 2 | 2 | 2 | 2 | 0 | 2 | 2 | 0 | NA | NA | NA | NA | 12 |
| Elbers | 2 | 2 | 2 | 2 | 0 | 2 | 2 | 1 | NA | NA | NA | NA | 13 |
| Barroso-Sousa | 2 | 1 | 2 | 2 | 0 | 0 | 0 | 1 | NA | NA | NA | NA | 8 |
| NCT(03102242) | 2 | 2 | 2 | 2 | 0 | 2 | 2 | 0 | NA | NA | NA | NA | 12 |
| NCT(04081688) | 2 | 2 | 2 | 2 | 0 | 2 | 2 | 0 | NA | NA | NA | NA | 12 |
| NCT(03421652) | 2 | 2 | 2 | 2 | 0 | 2 | 2 | 0 | NA | NA | NA | NA | 12 |
| Wise-Draper | 2 | 2 | 2 | 2 | 0 | 2 | 2 | 1 | 0 | 2 | 2 | 0 | 17 |
| Jabbour | 2 | 2 | 2 | 2 | 0 | 2 | 2 | 0 | 0 | 2 | 2 | 2 | 18 |
| Xie | 2 | 2 | 2 | 2 | 0 | 1 | 2 | 0 | 0 | 2 | 2 | 2 | 17 |
| NCT(02659540) | 2 | 2 | 2 | 2 | 0 | 2 | 2 | 0 | 0 | 2 | 2 | 2 | 18 |
