# Supplementary material for: Respiratory adverse effects in patients treated with immune checkpoint inhibitors in combination with radiotherapy: a systematic review and meta-analysis
Source: Radiat Oncol. 2024 Oct 1;19:134. doi: 10.1186/s13014-024-02489-4 (PMC11445955; doi:10.1186/s13014-024-02489-4)

Supplementary Figure 1. Forest plot of incidence rate of grades 3–5 cough in Patients Treated with Immune Checkpoint Inhibitors in Combination with Radiotherapy


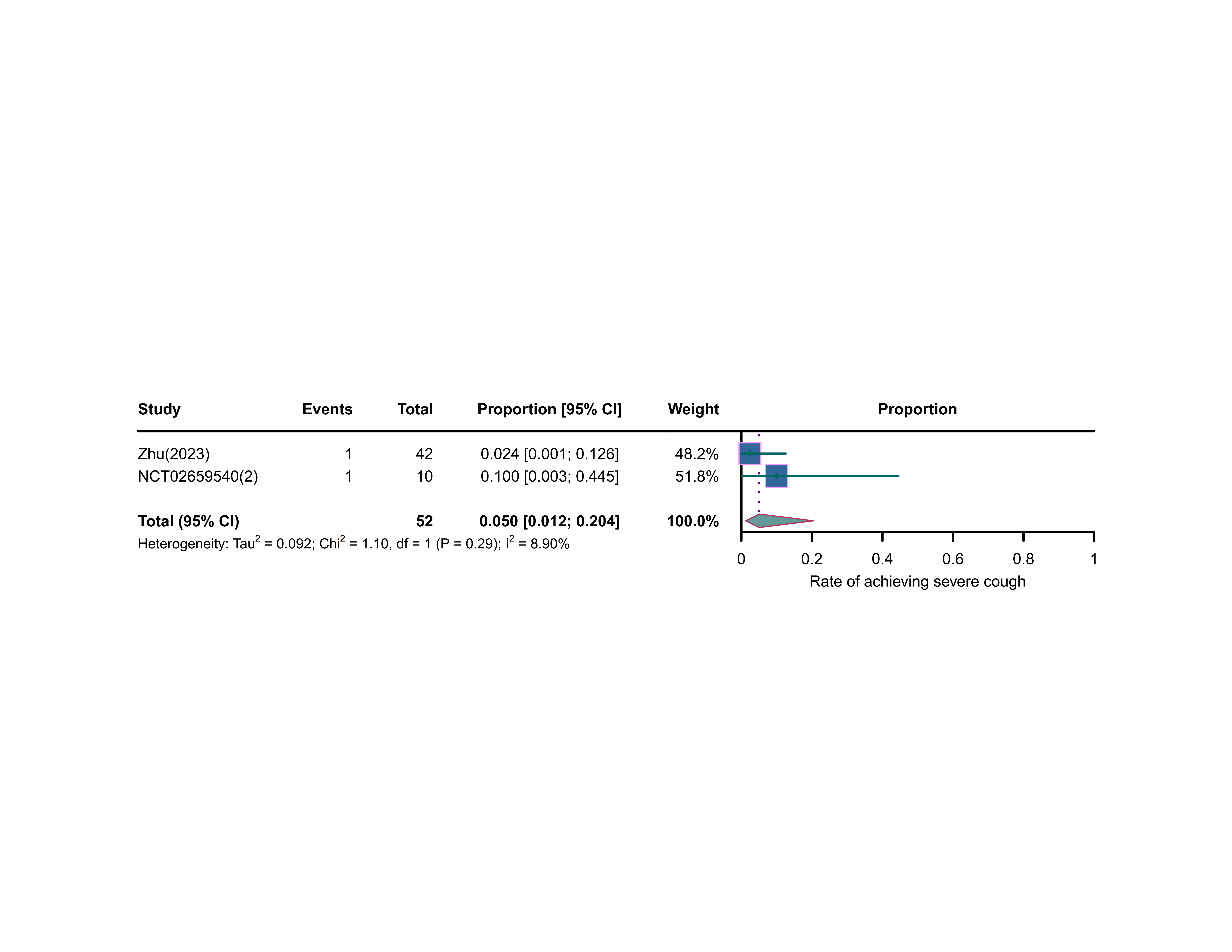


Supplementary Figure 2. Forest plot of incidence rate of grades 3–5 pneumonitis in Patients Treated with Immune Checkpoint Inhibitors in Combination with Radiotherapy


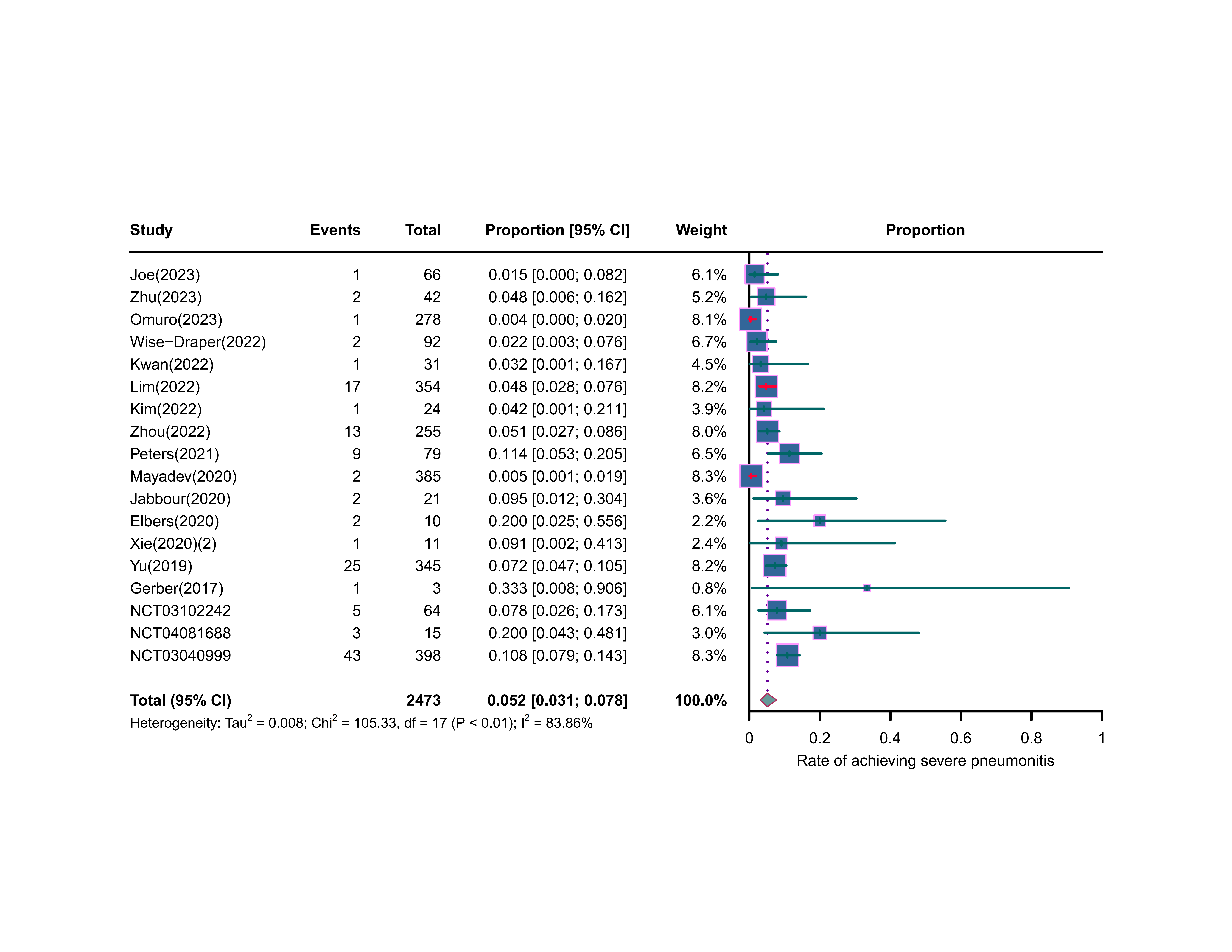


Supplementary Figure 3. Forest plot of incidence rate of grades 3–5 upper respiratory tract infection in Patients Treated with Immune Checkpoint Inhibitors in Combination with Radiotherapy


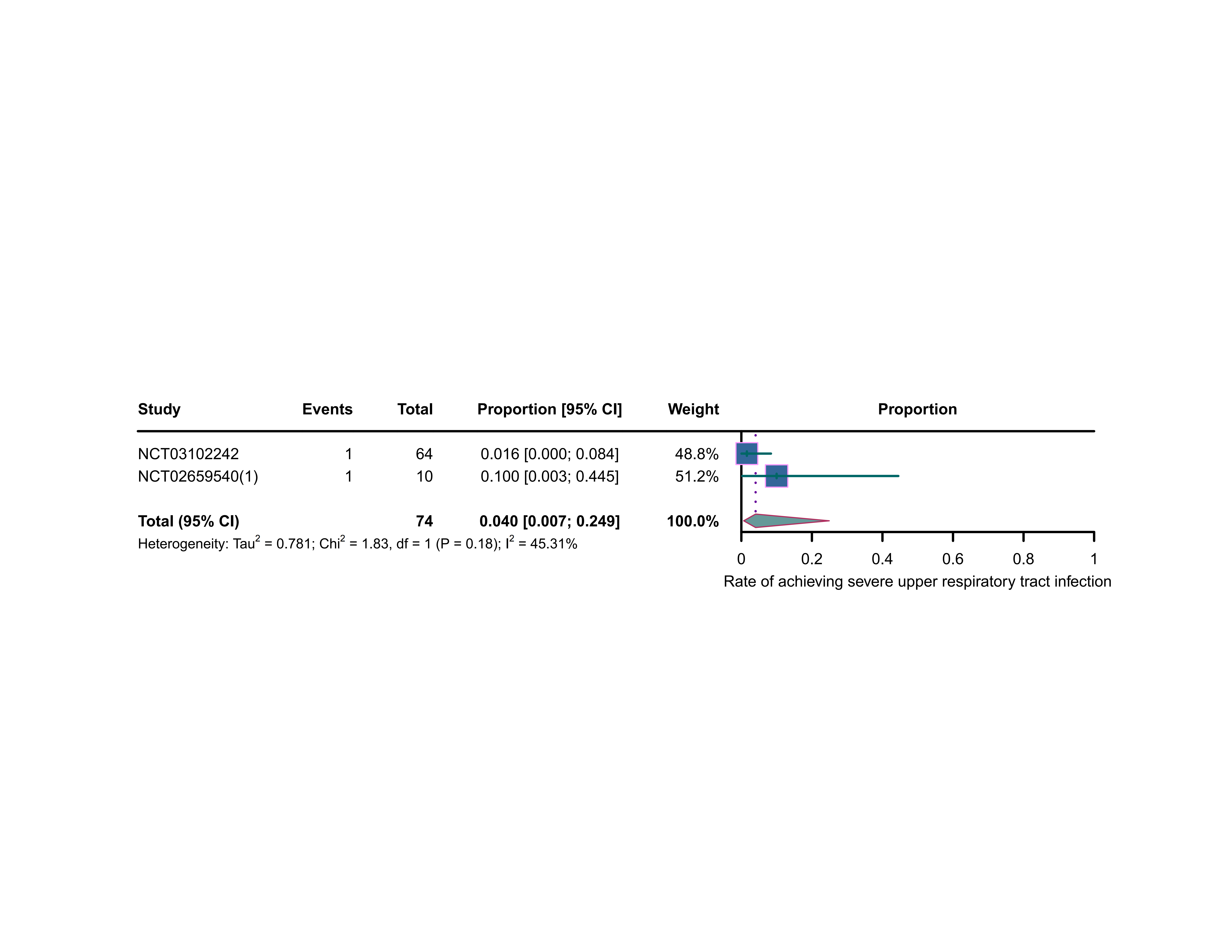


Supplementary Figure 4. Forest plot of incidence rate of grades 3–5 dyspnea in Patients Treated with Immune Checkpoint Inhibitors in Combination with Radiotherapy


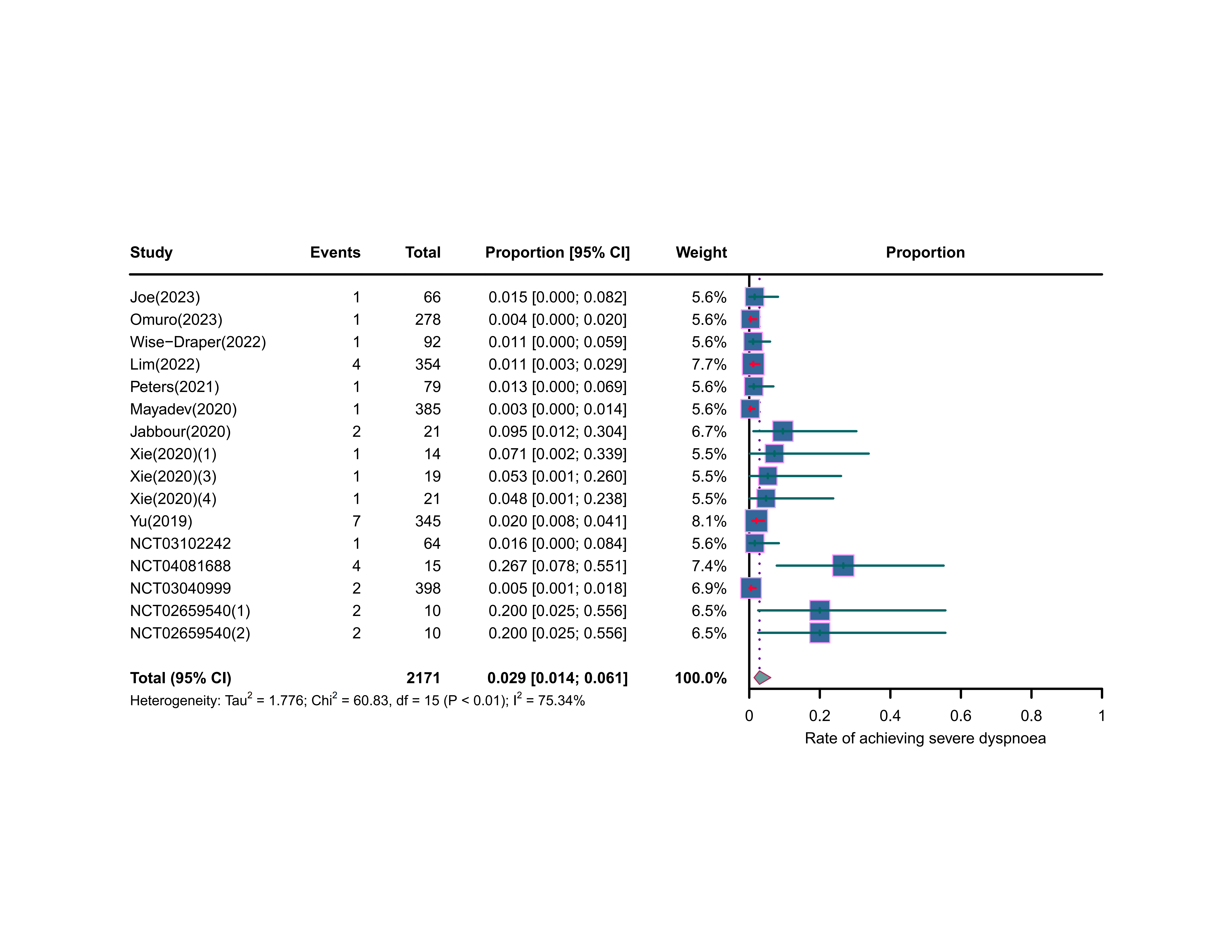


Supplementary Figure 5. Forest plot of incidence rate of grades 3–5 pneumonitis for subgroup analysis by different immune checkpoint inhibitors therapies


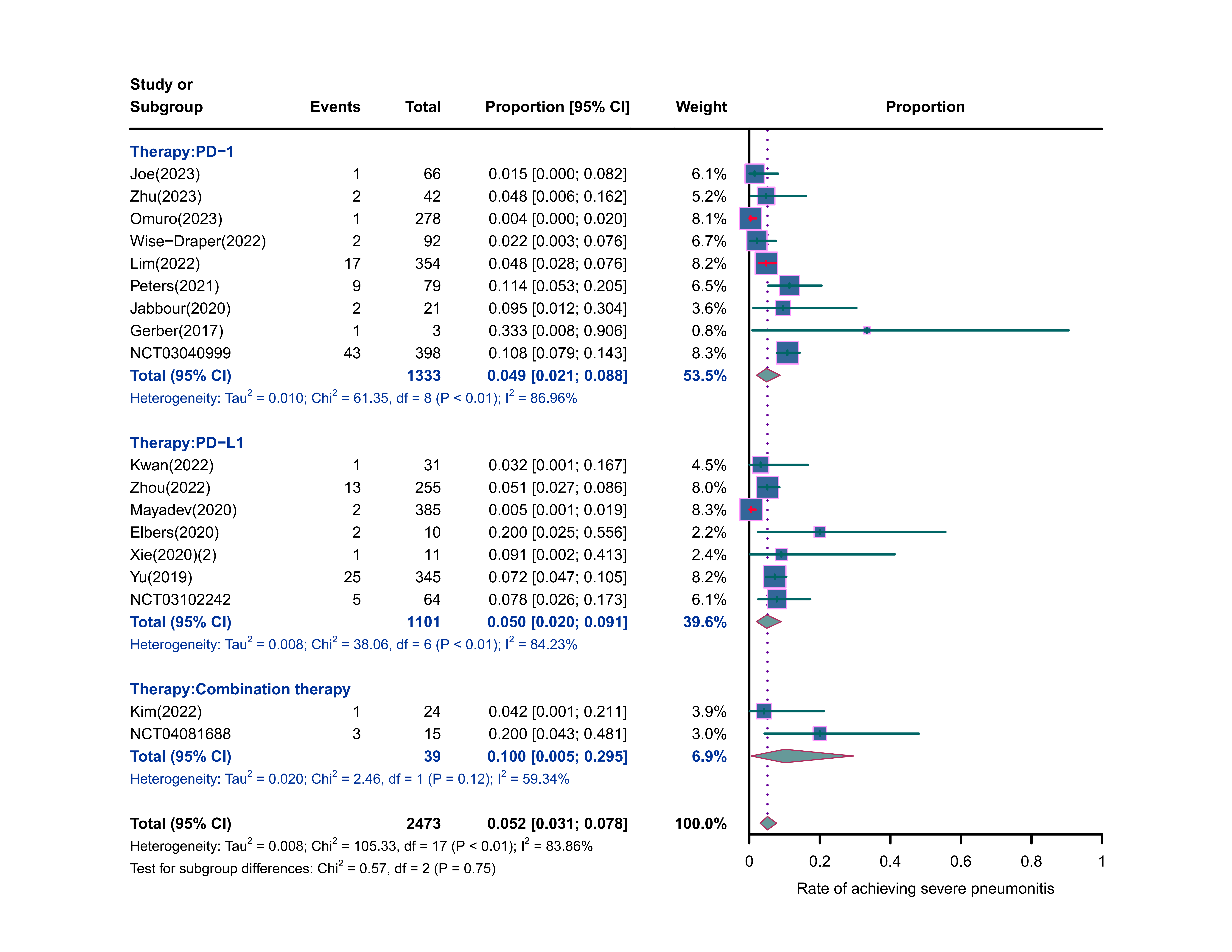


Supplementary Figure 6. Forest plot of incidence rate of grades 3–5 pneumonitis for subgroup analysis by different radiotherapies


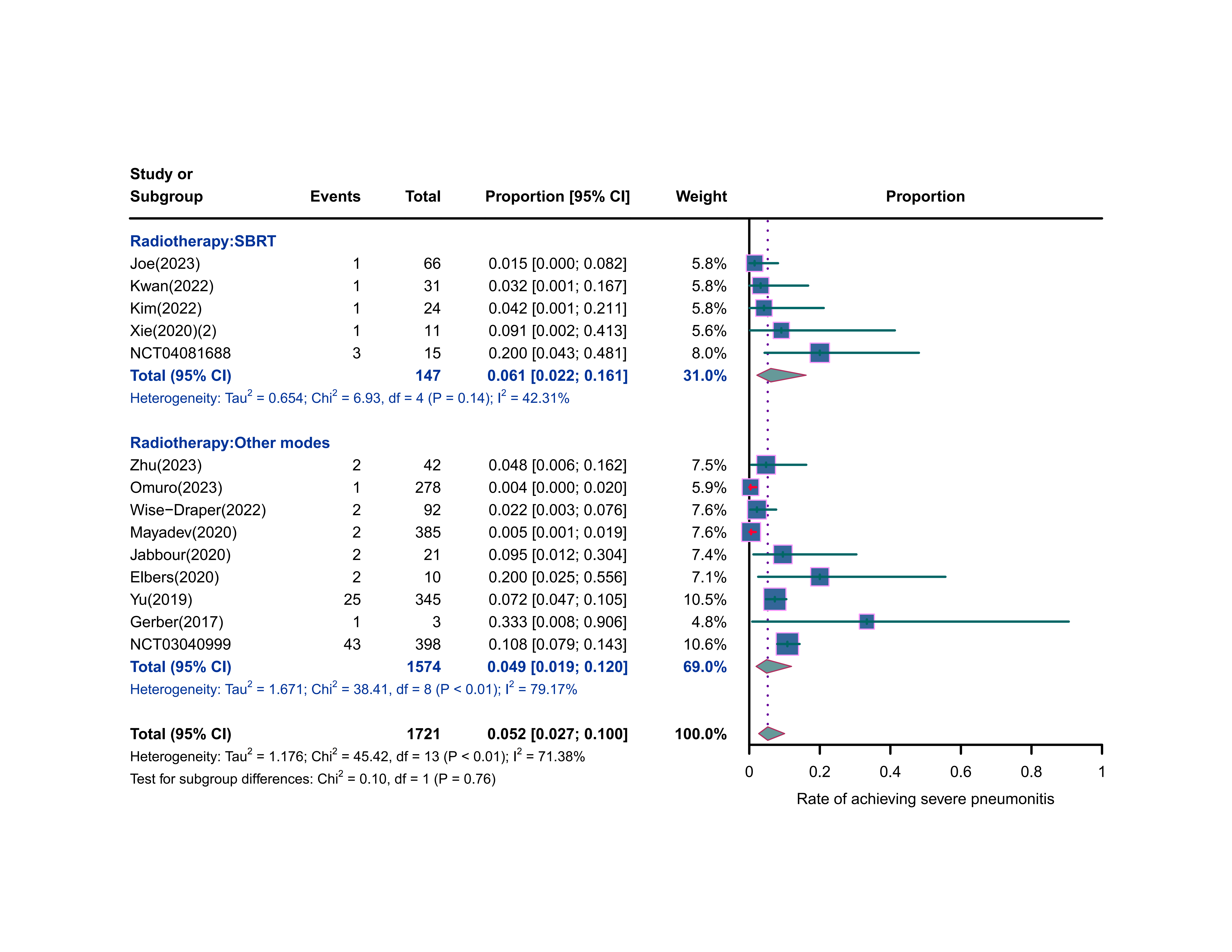


Supplementary Figure 7. Forest plot of incidence rate of grades 3–5 pneumonitis for subgroup analysis by different tumor locations


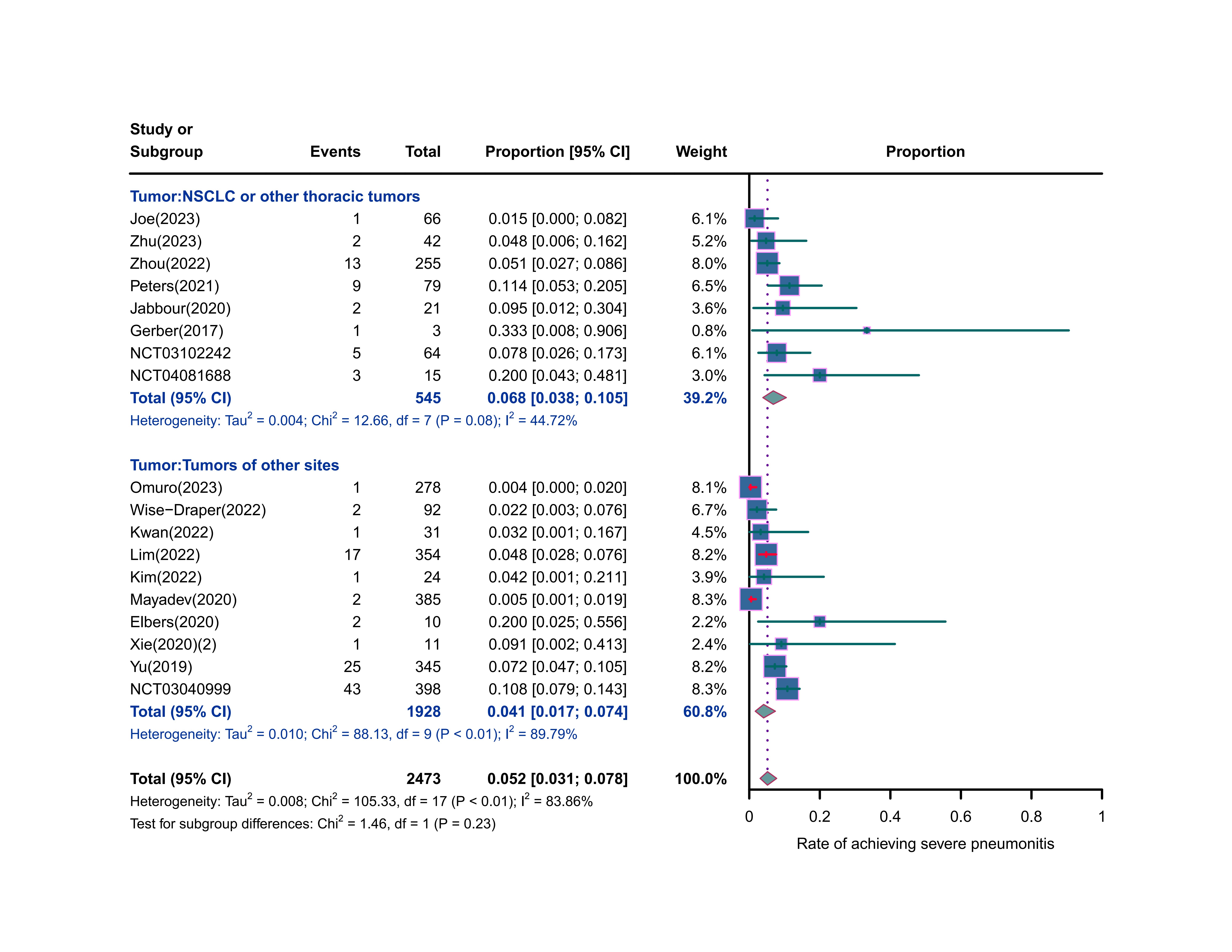


Supplementary Figure 8. Forest plot of incidence rate of grades 3–5 pneumonitis for subgroup analysis by different tumor locations


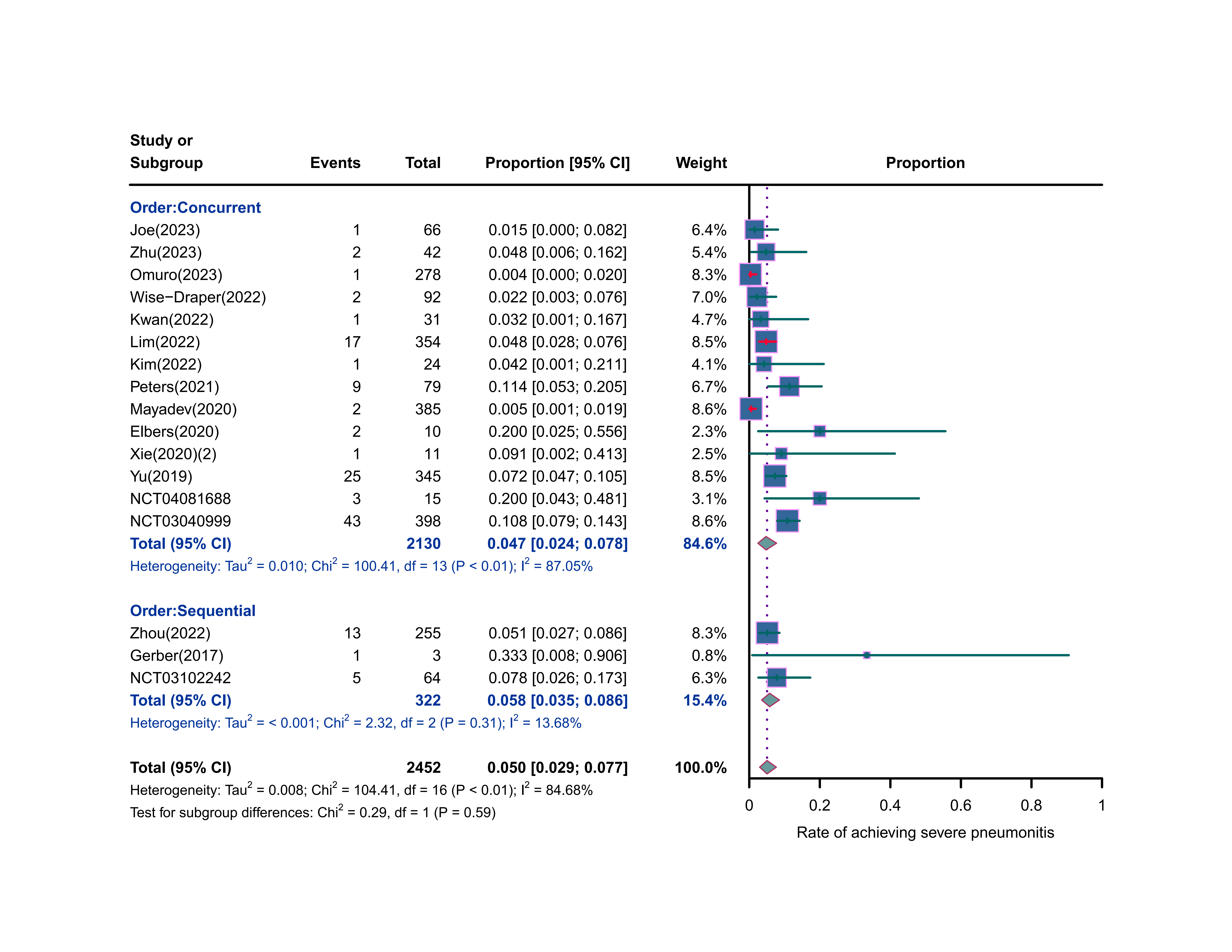


Supplementary Figure 9.Assessment of risk of bias for included Randomized Clinical Trials (RCTs) to determine the quality of included RCTs using the Cochrane risk of bias method.


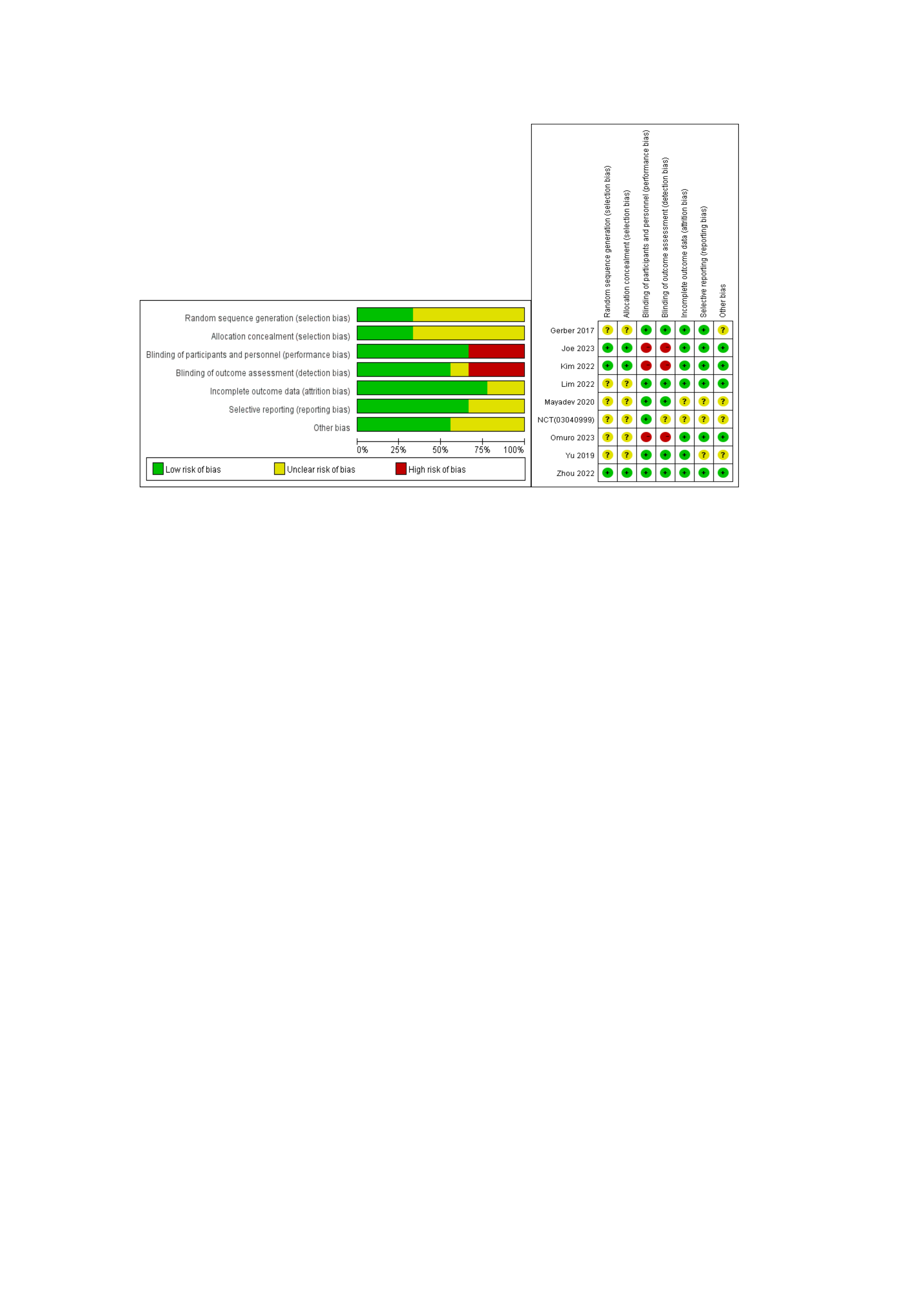


Supplementary Figure 10. Funnel plot of the incidence of cough.

Supplementary Figure 11. Funnel plot of the incidence of dyspnoea.

Supplementary Figure 12. Funnel plot of the incidence of upper respiratory tract infection.

Supplementary Figure 13. Funnel plot of the incidence of pneumonitis.

Supplementary Figure 14. Funnel plot of the incidence of severe dyspnoea.

Supplementary Figure 15. Funnel plot of the incidence of severe pneumonitis.

Supplementary Figure 16. Egger’s and Begg’s tests of the incidence of cough (begg: p=0.0561, egger: p=0.0739).


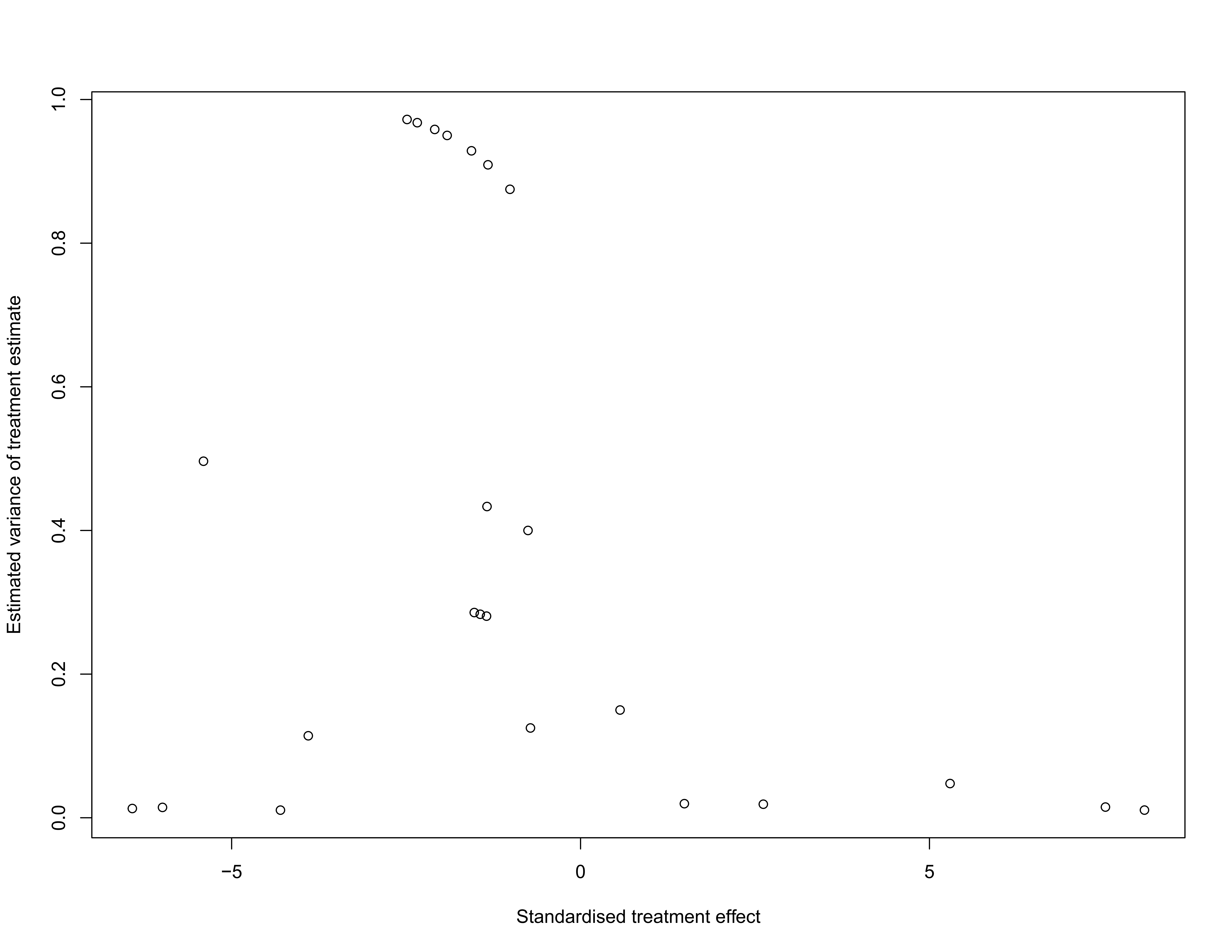


Supplementary Figure 17. Egger’s and Begg’s tests of the incidence of dyspnoea (begg: p=0.6519, egger: p＜0.0001).


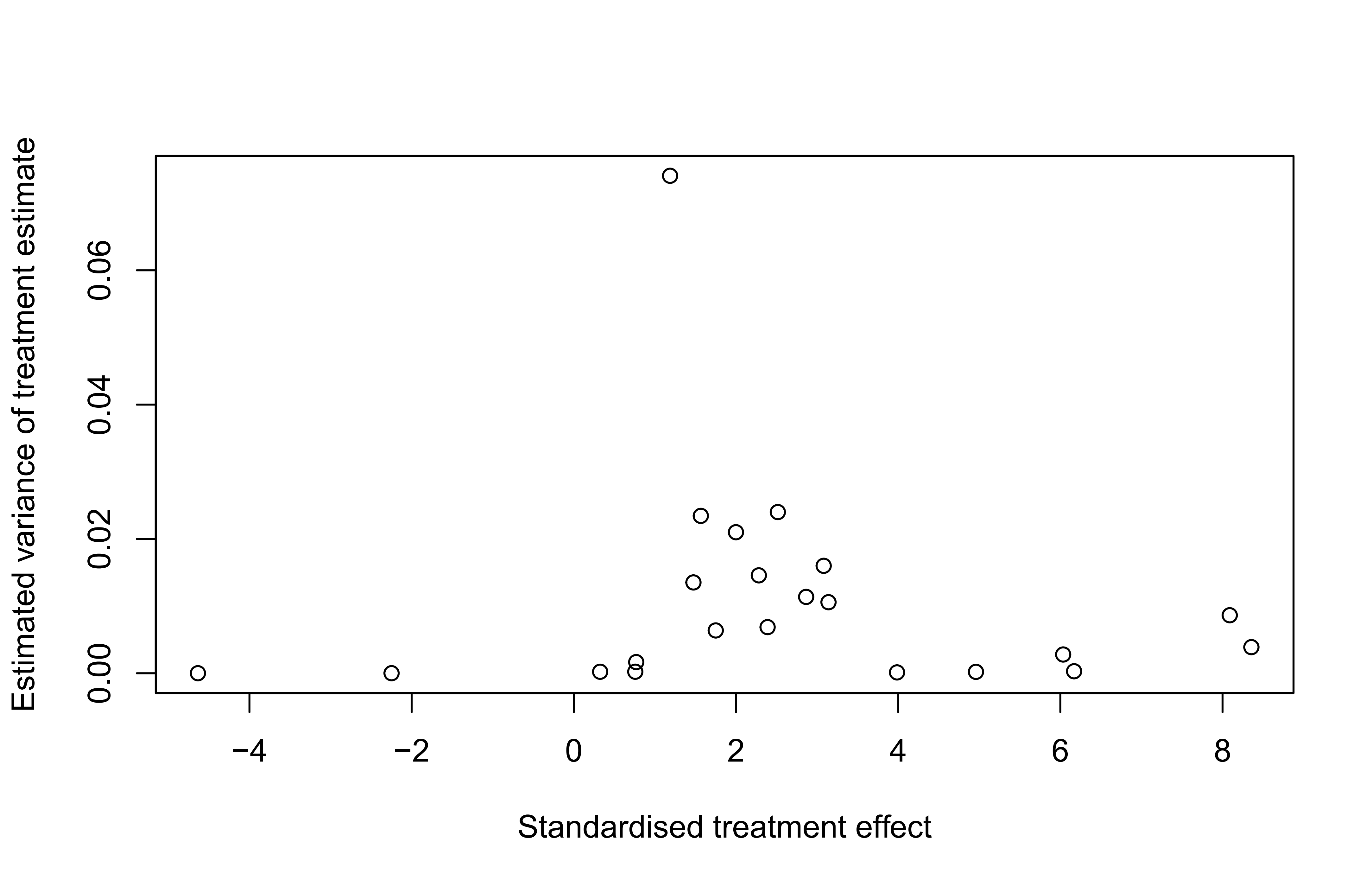


Supplementary Figure 18. Egger’s and Begg’s tests of the incidence of upper respiratory tract infection (begg: p=0.0763, egger: p=0.1224).


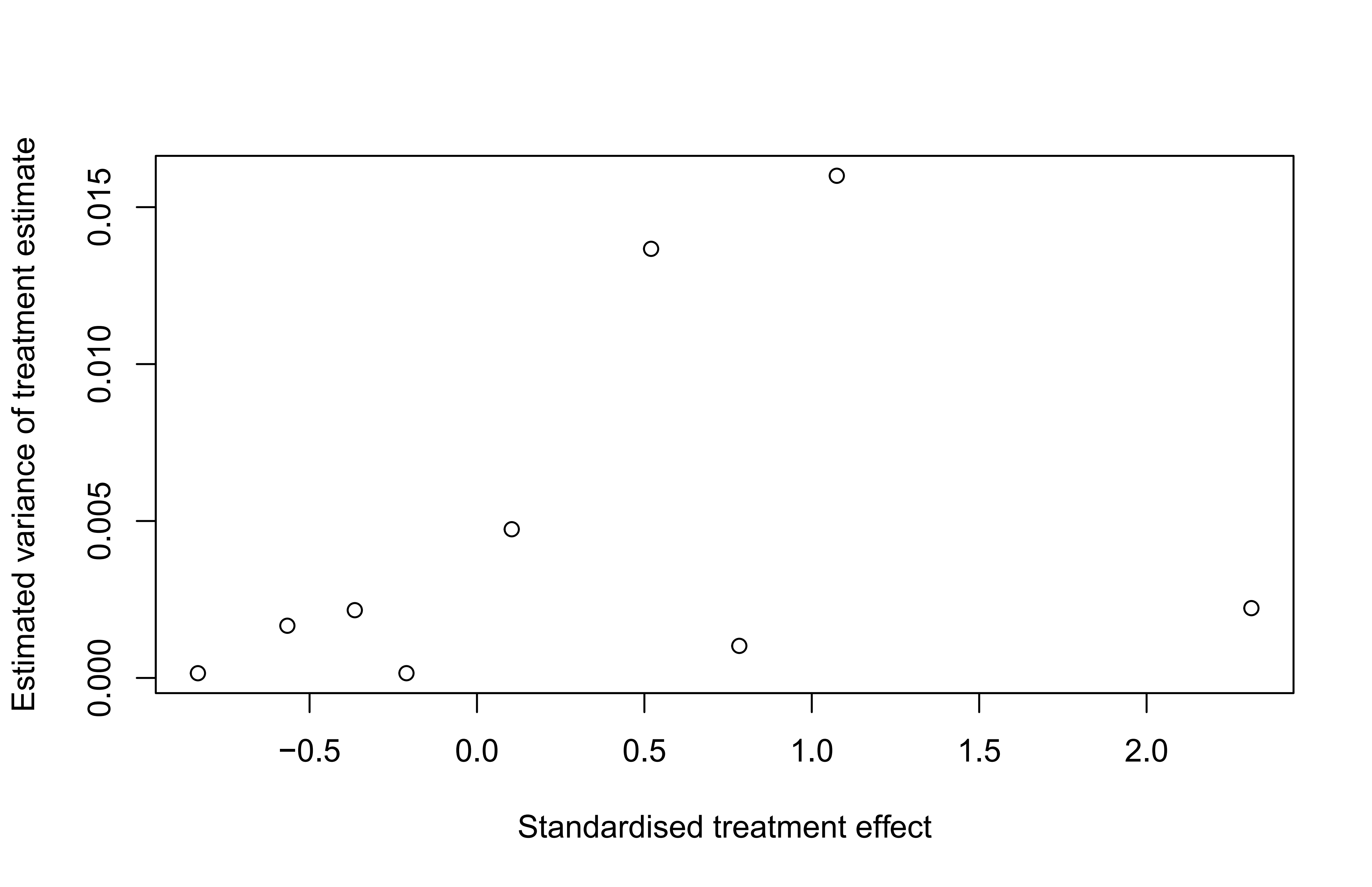


Supplementary Figure 19. Egger’s and Begg’s tests of the incidence of pneumonitis (begg: p=0.4419, egger: p=0.1710).


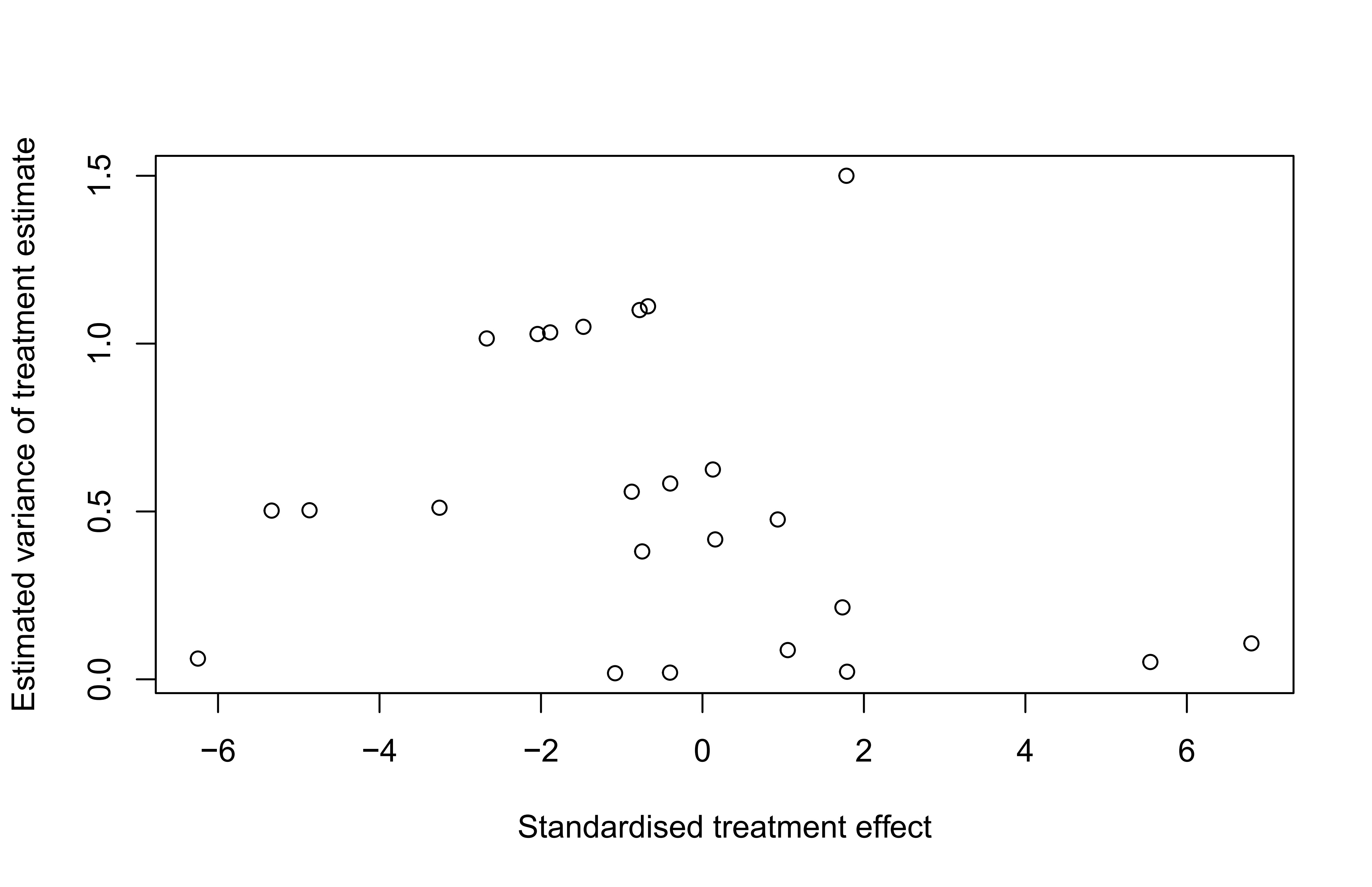


Supplementary Figure 20. Egger’s and Begg’s tests of the incidence of severe dyspnoea (begg: p=0.2789, egger: p=0.9195).


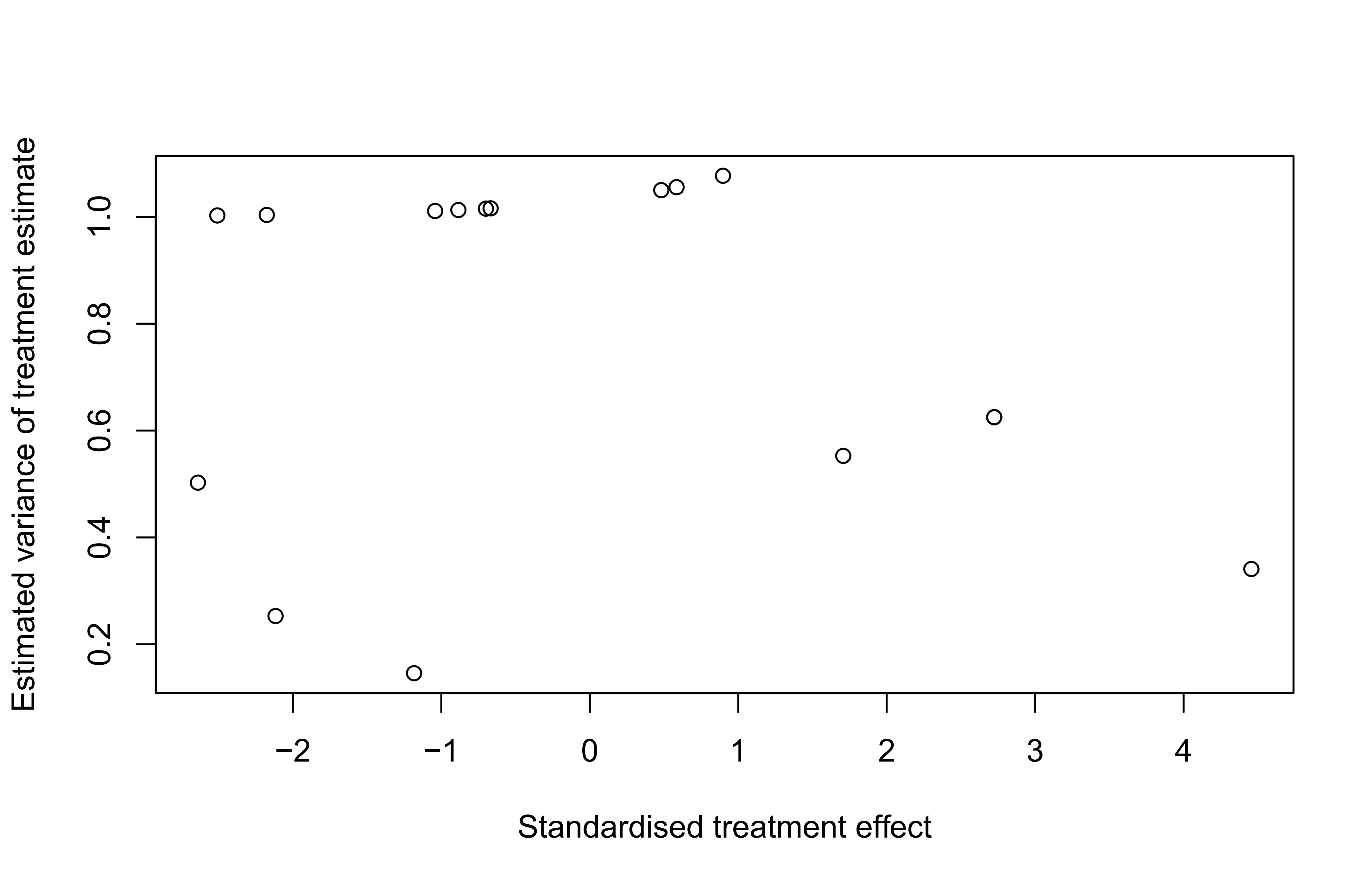


Supplementary Figure 21. Egger’s and Begg’s tests of the incidence of severe pneumonitis (begg: p=0.4047, egger: p=0.3665).


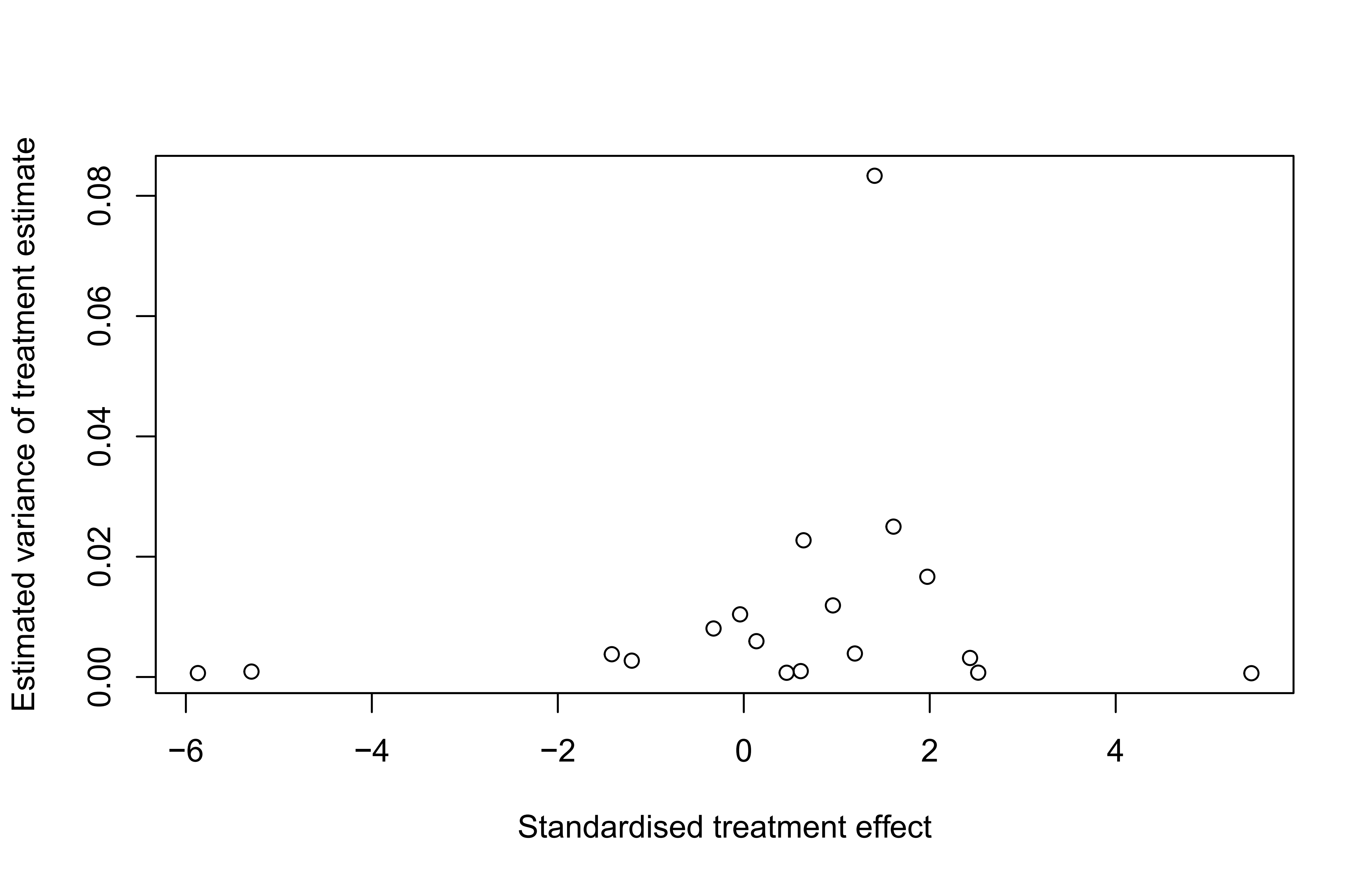


Supplementary Figure 22. Forest plot of the sensitivity analysis through the systematic elimination of individual studies. After the removal of each study, there were no significant deviations from the initial analytical outcomes of cough.

Supplementary Figure 23. Forest plot of the sensitivity analysis through the systematic elimination of individual studies. After the removal of each study, there were no significant deviations from the initial analytical outcomes of dyspnoea.

Supplementary Figure 24. Forest plot of the sensitivity analysis through the systematic elimination of individual studies. After the removal of each study, there were no significant deviations from the initial analytical outcomes of upper respiratory tract infection.

Supplementary Figure 25. Forest plot of the sensitivity analysis through the systematic elimination of individual studies. After the removal of each study, there were no significant deviations from the initial analytical outcomes of pneumonitis.

Supplementary Figure 26. Forest plot of the sensitivity analysis through the systematic elimination of individual studies. After the removal of each study, there were no significant deviations from the initial analytical outcomes of severe dyspnoea.

Supplementary Figure 27. Forest plot of the sensitivity analysis through the systematic elimination of individual studies. After the removal of each study, there were no significant deviations from the initial analytical outcomes of severe pneumonitis.

Supplementary Figure 28. Funnel plot and forest plot of the sensitivity analysis through the trim and fill method. The result showed that there were no significant deviations from the initial analytical outcomes of cough.


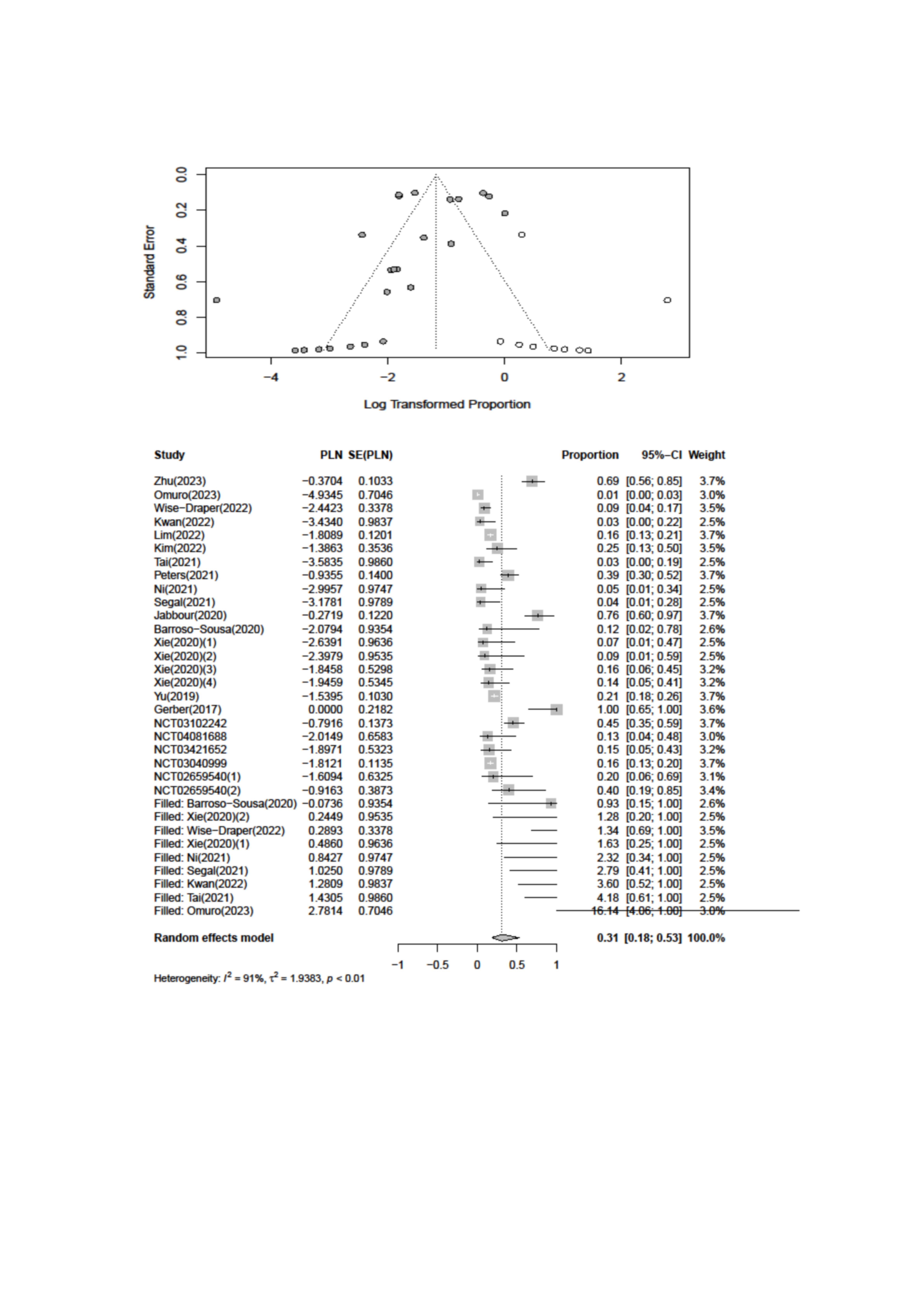


Supplementary Figure 29. Funnel plot and forest plot of the sensitivity analysis through the trim and fill method. The result showed that there were some discrepancies observed in the initial analytical outcomes of dyspnoea, and it demonstrated that the analysis of dyspnoea might be influenced by publication bias and heterogeneity.


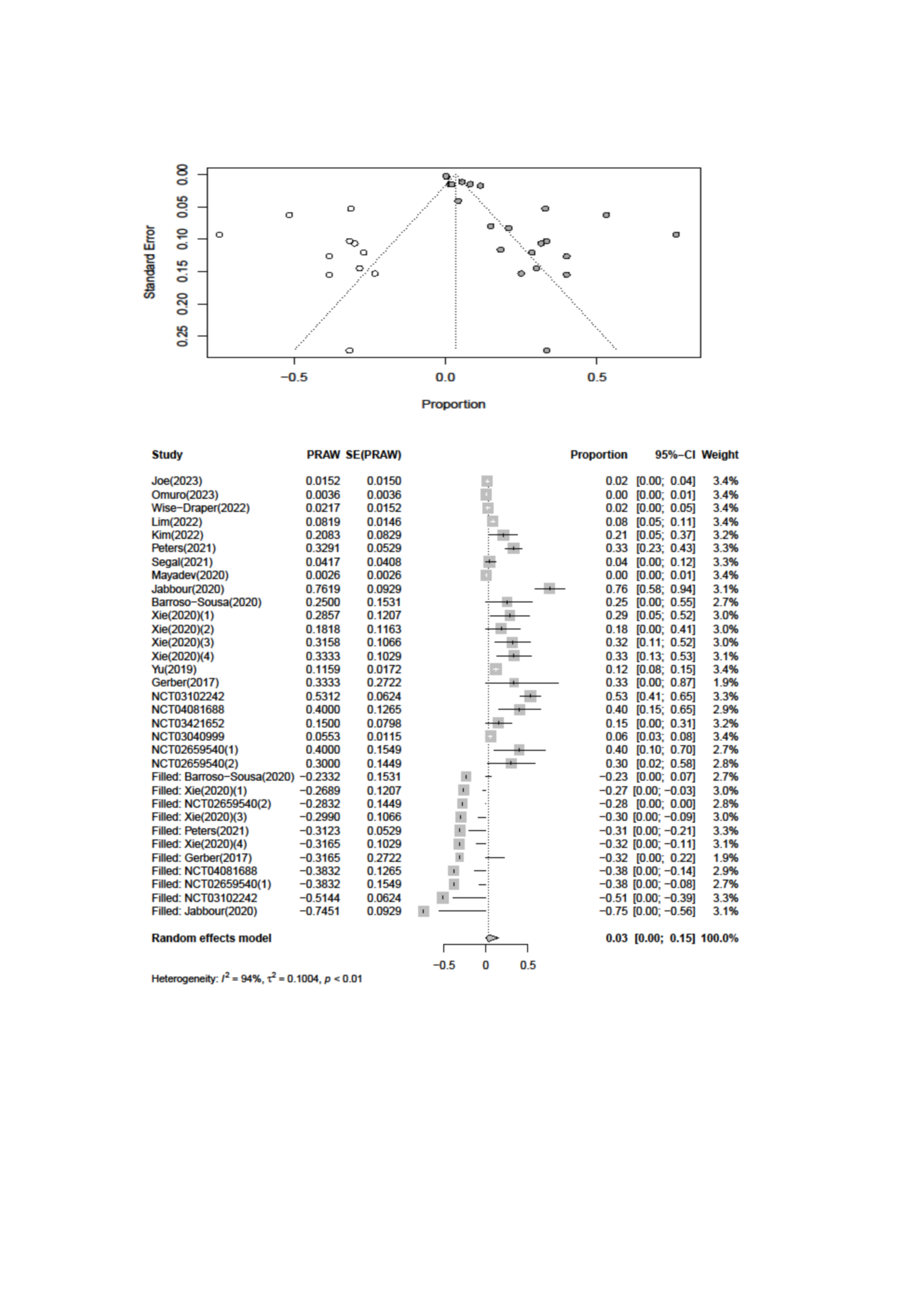


Supplementary Figure 30. Funnel plot and forest plot of the sensitivity analysis through the trim and fill method. The result showed that there were no significant deviations from the initial analytical outcomes of upper respiratory tract infection.


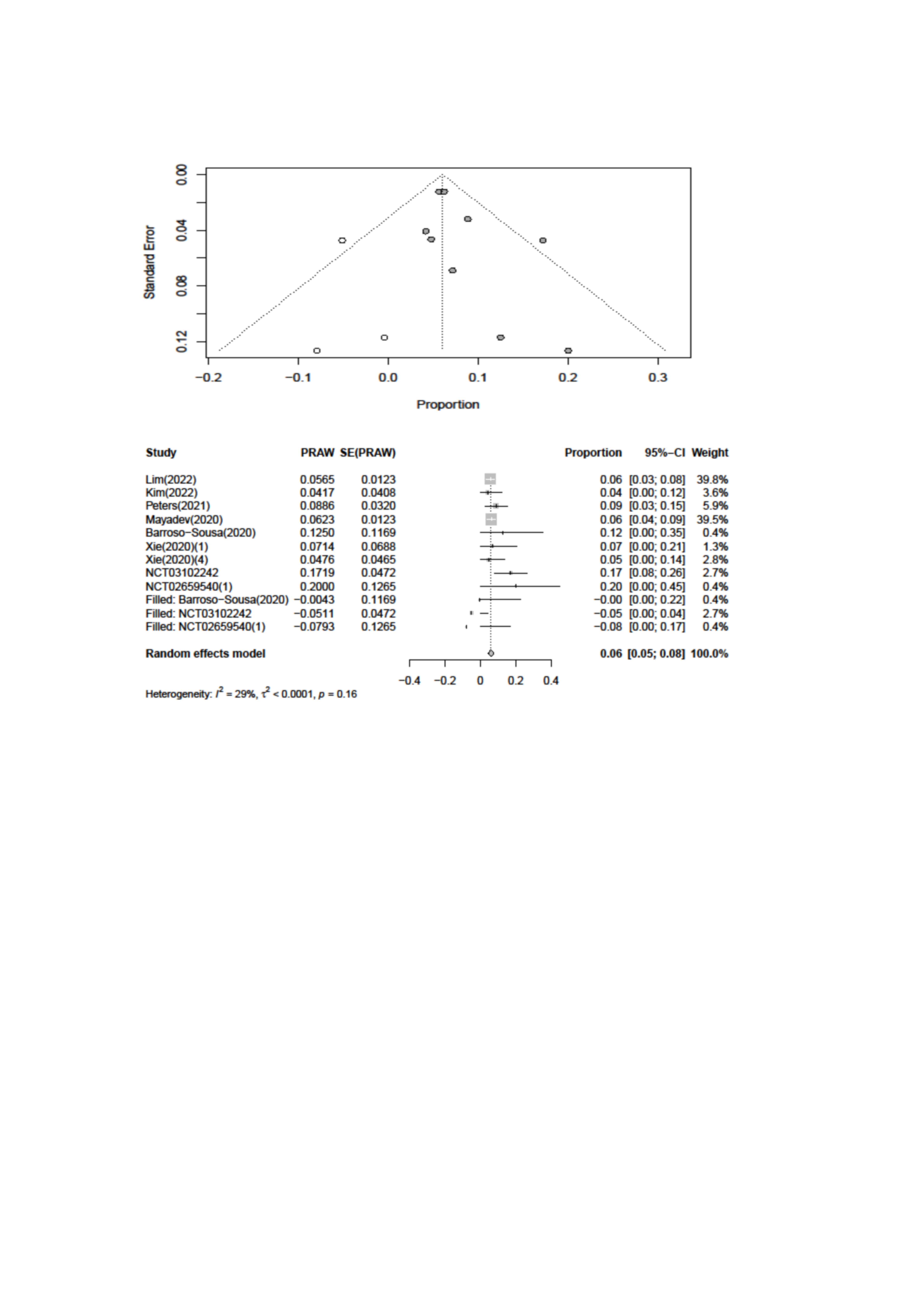


Supplementary Figure 31. Funnel plot and forest plot of the sensitivity analysis through the trim and fill method. The result showed that there were no significant deviations from the initial analytical outcomes of pneumonitis.


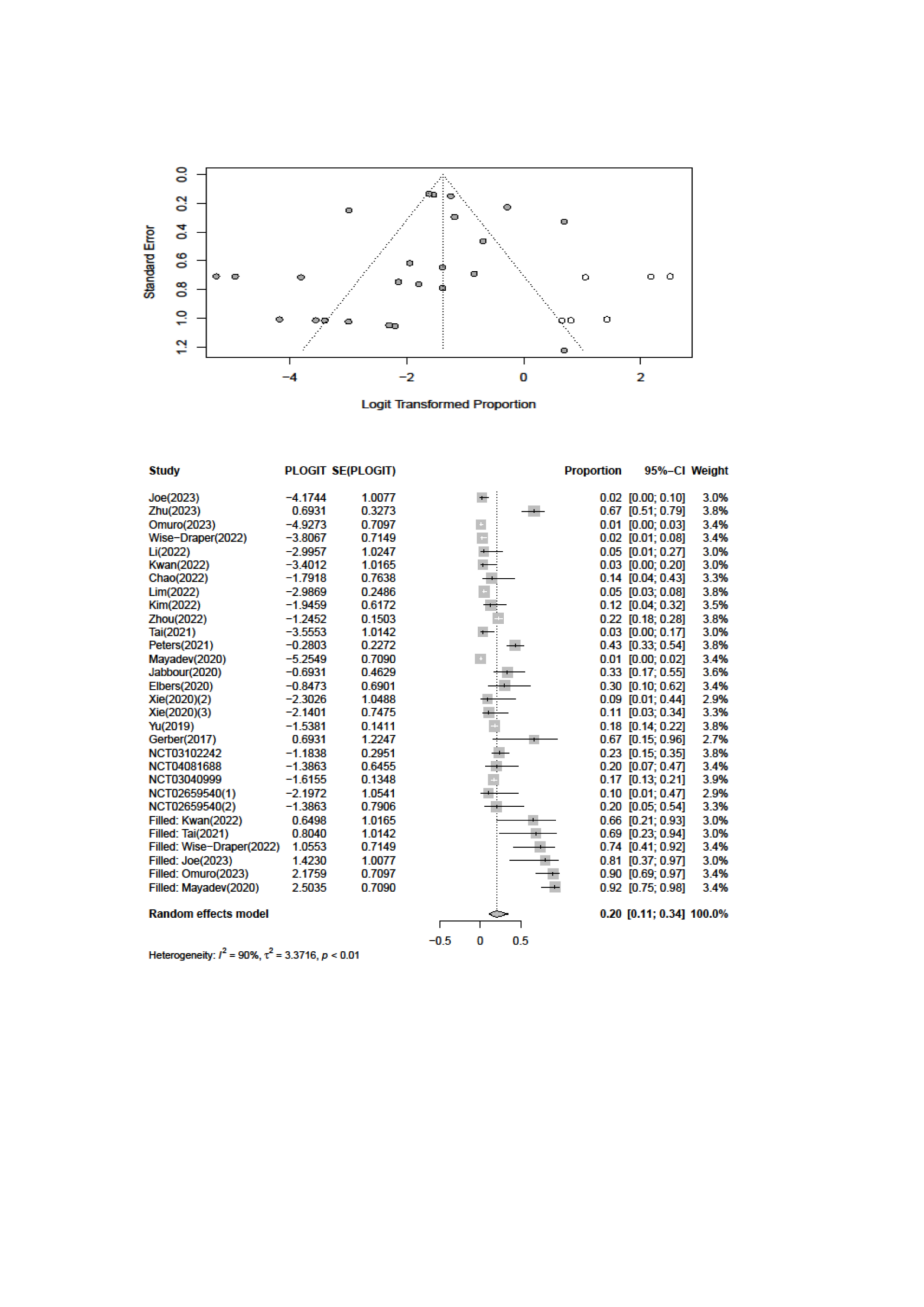


Supplementary Figure 32. Funnel plot and forest plot of the sensitivity analysis through the trim and fill method. The result showed that there were no significant deviations from the initial analytical outcomes of severe dyspnoea.


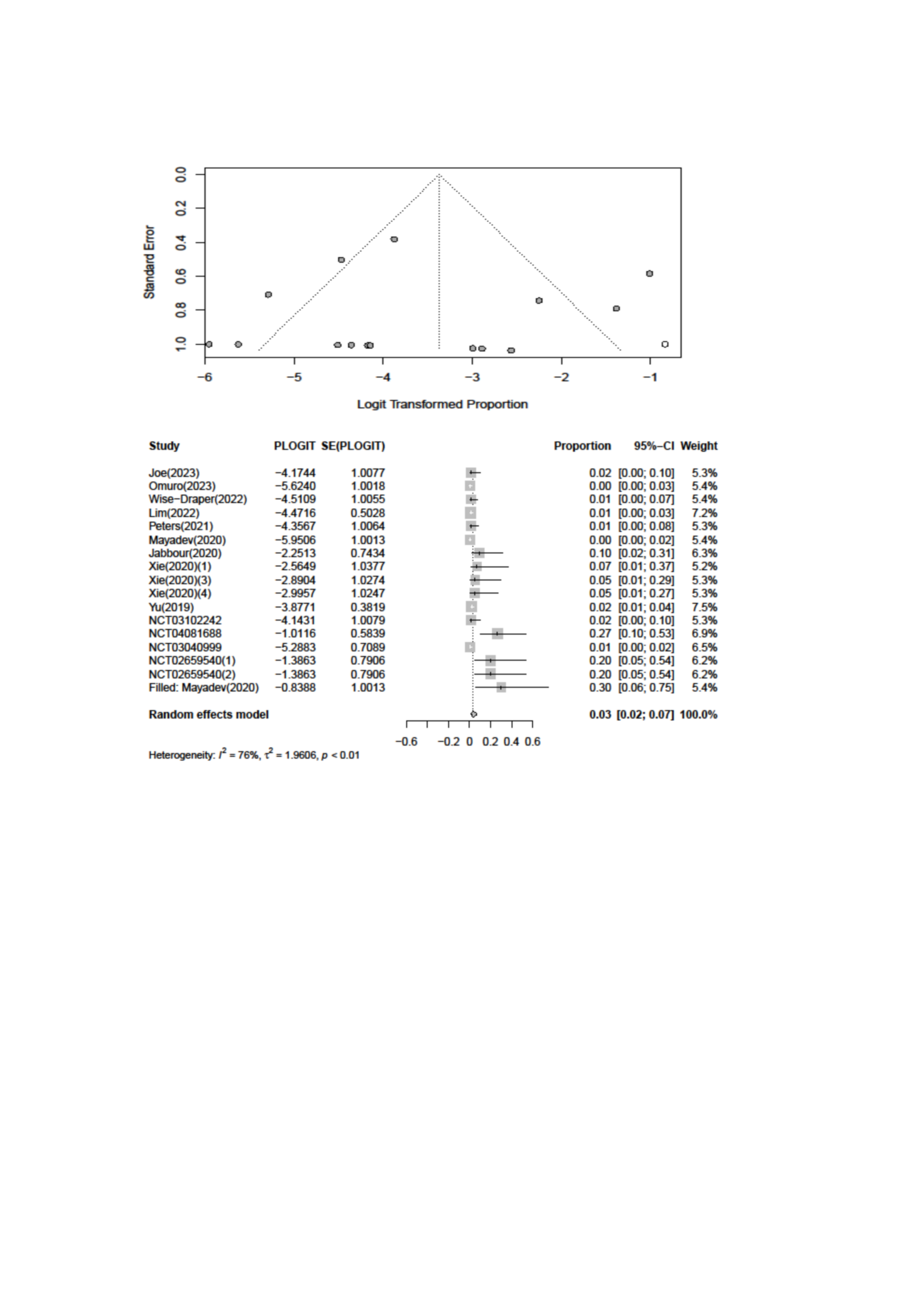


Supplementary Figure 32. Funnel plot and forest plot of the sensitivity analysis through the trim and fill method. The result showed that there were no significant deviations from the initial analytical outcomes of severe pneumonitis.


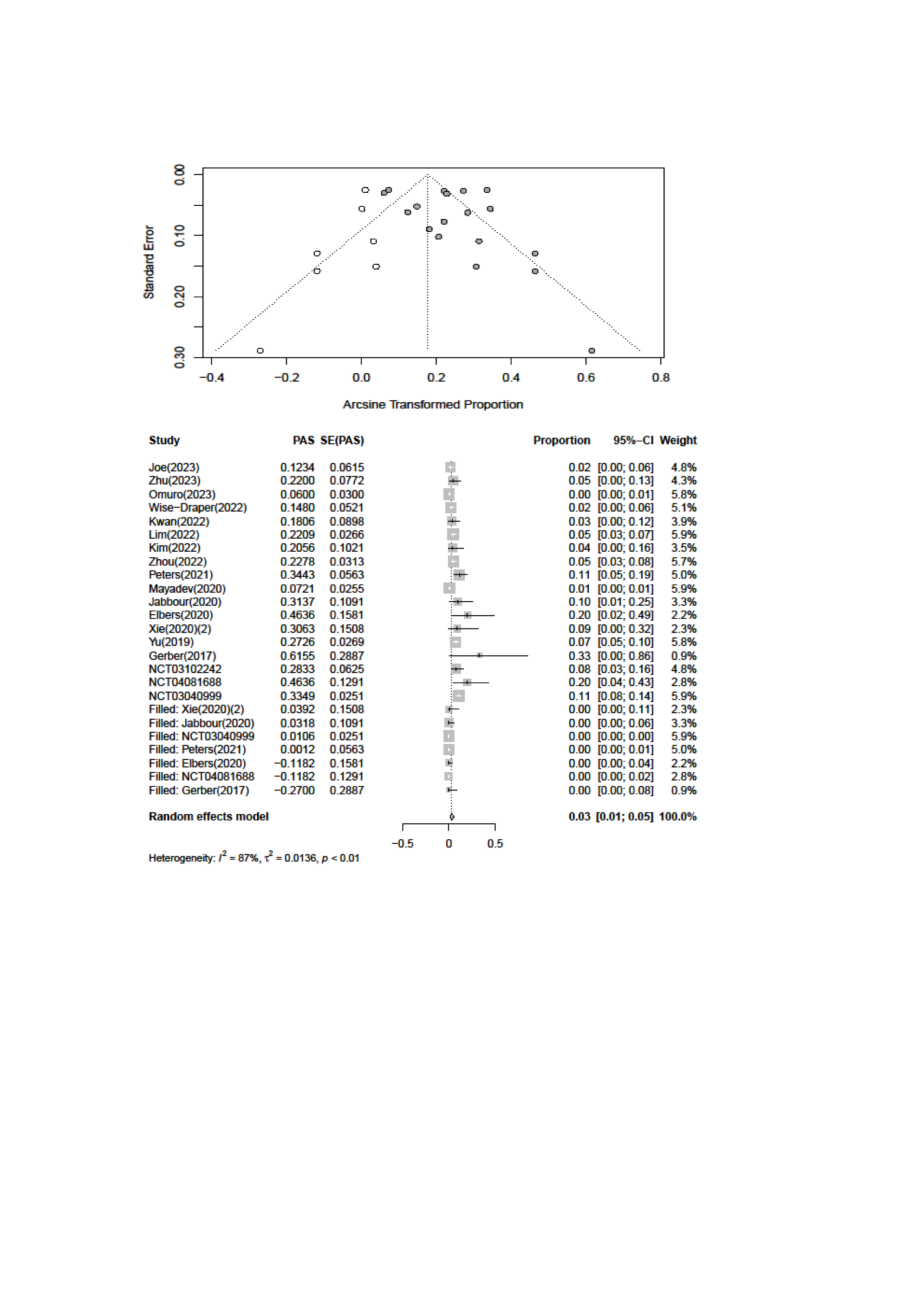

Supplement: Supplementary file 2 — Supplementary Material 2 [file 13014_2024_2489_MOESM2_ESM.doc]
